# PDGFRβ promotes oncogenic progression via STAT3/STAT5 hyperactivation in anaplastic large cell lymphoma

**DOI:** 10.1186/s12943-022-01640-7

**Published:** 2022-08-31

**Authors:** I. Garces de los Fayos Alonso, L. Zujo, I. Wiest, P. Kodajova, G. Timelthaler, S. Edtmayer, M. Zrimšek, S. Kollmann, C. Giordano, M. Kothmayer, H. A. Neubauer, S. Dey, M. Schlederer, B. S. Schmalzbauer, T. Limberger, C. Probst, O. Pusch, S. Högler, S. Tangermann, O. Merkel, A. I. Schiefer, C. Kornauth, N. Prutsch, M. Zimmerman, B. Abraham, J. Anagnostopoulos, L. Quintanilla-Martinez, S. Mathas, P. Wolf, D. Stoiber, P. B. Staber, G. Egger, W. Klapper, W. Woessmann, T. A. Look, P. Gunning, S. D. Turner, R. Moriggl, S. Lagger, L. Kenner

**Affiliations:** 1 Department of Pathology, Medical University of Vienna, 1090 Vienna, Austria; 2 Unit of Laboratory Animal Pathology, University of Veterinary Medicine Vienna, 1210 Vienna, Austria; 3 Division of Nuclear Medicine, Medical University of Vienna, 1090 Vienna, Austria; 4 Center for Cancer Research, Medical University of Vienna, 1090 Vienna, Austria; 5 Division Pharmacology, Department of Pharmacology, Physiology and Microbiology, Karl Landsteiner University of Health Sciences, 3500 Krems, Austria; 6 Institute of Pharmacology and Toxicology, University of Veterinary Medicine Vienna, 1210 Vienna, Austria; 7 Centre for Anatomy and Cell Biology, Medical University of Vienna, 1090 Vienna, Austria; 8 Institute of Animal Breeding and Genetics, Unit of Functional Cancer Genomics, University of Veterinary Medicine Vienna, 1210 Vienna, Austria; 9 Department of Dermatology, Medical University of Graz, 8036 Graz, Austria; 10 Center for Medical Research (ZMF), Medical University of Graz, 8010 Graz, Austria; 11 CBMed Core Lab, Medical University of Vienna, 1090 Vienna, Austria; 12 Department of Medicine I, Division of Hematology and Hemostaseology, Medical University of Vienna, 1090 Vienna, Austria; 13 Comprehensive Cancer Center Vienna, Vienna General Hospital, Medical University of Vienna, 1090 Vienna, Austria; 14 Department of Pediatric Oncology, Dana-Farber Cancer Institute, Harvard Medical School, Boston, MA USA; 15 Department of Computational Biology, St. Jude Children’s Research Hospital, Memphis, TN USA; 16 Institute of Pathology, University of Wuerzburg, 97080 Würzburg, Germany; 17 Institute of Pathology, Charité-Medical University of Berlin, 10117 Berlin, Germany; 18 Institute of Pathology and Neuropathology and Cluster of excellence iFIT, “Image-Guided and Functionally Instructed Tumor Therapy”, University of Tübingen, 72076 Tübingen, Germany; 19 Department of Hematology, Oncology, and Cancer Immunology, Charité-Medical University of Berlin, 12200 Berlin, Germany; 20 German Cancer Consortium (DKTK) German Cancer Research Center (DKFZ), 69120 Heidelberg, Germany; 21 Max-Delbrück-Center (MDC) for Molecular Medicine, 13125 Berlin, Germany; 22 Experimental and Clinical Research Center, a joint cooperation between the Charité and the MDC, 13125 Berlin, Germany; 23 Boltzmann Institute Applied Diagnostics, 1090 Vienna, Austria; 24 Department of Pathology, Hematopathology Section and Lymph Node Registry, University of Kiel/University Hospital Schleswig-Holstein, 24105 Kiel, Germany; 25 Pediatric Hematology and Oncology, University Hospital Hamburg-Eppendorf, Hamburg, Germany; 26 Department of Chemical and Physical Sciences, University of Toronto Mississauga, Mississauga, ON L5L 1C6 Canada; 27 Department of Chemistry, University of Toronto, Toronto, ON M5S 3H6 Canada; 28 Division of Cellular and Molecular Pathology, Department of Pathology, University of Cambridge, Cambridge, CB20QQ UK; 29 Central European Institute of Technology (CEITEC), Masaryk University, Brno, Czech Republic; 30 Christian Doppler Laboratory of Applied Metabolomics, Department of Biomedical Imaging and Image-guided Therapy, Medical University of Vienna, 1090 Vienna, Austria

**Keywords:** ALCL, PDGFRβ, STAT3, STAT5A, STAT5B, NPM-ALK, Apoptosis

## Abstract

**Background:**

Anaplastic large cell lymphoma (ALCL) is an aggressive non-Hodgkin T cell lymphoma commonly driven by NPM-ALK. AP-1 transcription factors, cJUN and JUNb, act as downstream effectors of NPM-ALK and transcriptionally regulate PDGFRβ. Blocking PDGFRβ kinase activity with imatinib effectively reduces tumor burden and prolongs survival, although the downstream molecular mechanisms remain elusive.

**Methods and results:**

In a transgenic mouse model that mimics PDGFRβ-driven human ALCL in vivo, we identify PDGFRβ as a driver of aggressive tumor growth. Mechanistically, PDGFRβ induces the pro-survival factor Bcl-x_L_ and the growth-enhancing cytokine IL-10 via STAT5 activation. CRISPR/Cas9 deletion of both STAT5 gene products, STAT5A and STAT5B, results in the significant impairment of cell viability compared to deletion of STAT5A, STAT5B or STAT3 alone. Moreover, combined blockade of STAT3/5 activity with a selective SH2 domain inhibitor, AC-4-130, effectively obstructs tumor development in vivo.

**Conclusions:**

We therefore propose PDGFRβ as a novel biomarker and introduce PDGFRβ-STAT3/5 signaling as an important axis in aggressive ALCL. Furthermore, we suggest that inhibition of PDGFRβ or STAT3/5 improve existing therapies for both previously untreated and relapsed/refractory ALK^+^ ALCL patients.

**Supplementary Information:**

The online version contains supplementary material available at 10.1186/s12943-022-01640-7.

## Key points

- PDGFRβ-STAT5 activity, acting in parallel to the NPM-ALK-STAT3 signaling axis, correlates with an inferior Event Free Survival (EFS) and Cumulative Incidence of Relapse (CI-R) in ALCL.
- PDGFRβ induced STAT5 activity, increases proliferation by stimulating IL-10 secretion and blocking apoptosis by upregulating Bcl-x_L_, characterizing PDGFRβ as a novel biomarker.
- Combined loss of STAT5A and STAT5B is lethal in ALK^+^ ALCL, suggesting STAT5 as a valid therapeutic target that can be successfully inhibited with the STAT inhibitor AC-4-130.

## Introduction

Anaplastic Large Cell Lymphoma (ALCL), characterized by expression of CD30, refers to a family of Non-Hodgkin T cell lymphomas divided into four entities: systemic Anaplastic Lymphoma Kinase (ALK) positive (ALK^+^ ALCL), systemic ALK negative (ALK^−^ ALCL), primary cutaneous ALCL (pc-ALCL) and breast-implant associated (BIA-ALCL) [1]. In 70% of systemic ALK^+^ ALCL cases, patients present with the *t(2;5)(p23;35)* translocation, resulting in abundant cytoplasmic and nuclear expression of the Nucleophosmin-Anaplastic Lymphoma Kinase (NPM-ALK) chimeric protein [2]. The expression of this ligand-independent oncogene is mediated via the *NPM1* promoter but drives disease pathogenesis via the ALK kinase domain [3]. NPM-ALK stimulates a plethora of oncogenic signal transduction pathways including JAK/STAT, RAS/RAF/ERK, PI3K/AKT/mTOR and JNK/p38/AP-1, but the key vulnerable nodes for targeting remain illusive [4]. In the context of ALCL, STAT3 has been identified as a crucial signaling modulator downstream of NPM-ALK responsible for disease maintenance [5–7]. Additional members of the STAT family, such as STAT1 [8] and STAT5A/B [9], have also been reported in ALCL however their function is not extensively studied and remains enigmatic.

Event Free Survival (EFS) and Overall Survival (OS) rates for ALK^+^ ALCL patients treated with standard CHOP (cyclophosphamide, doxorubicin, vincristine and prednisone) chemotherapy are relatively succesful [10–13]. However, current relapse rates range from 20 to 40%, highlighting the urgency to develop alternative therapy approaches for relapsed patients [14]. The restricted expression of ALK in neuronal cells during development make it an ideal drug target with potential to overcome relapse. First-generation ALK tyrosine kinase inhibitor (TKI) crizotinib (X*alkori*), initially approved by the Federal Drug Administration (FDA) for Non-Small Cell Lung Cancer (NSCLC) patients harboring ALK fusions, has recently been authorized for pediatric patients with relapsed or refractory systemic ALK^+^ ALCL (NCT00939770). Second [15–19], third [20], and fourth-generations [21] of ALK TKIs have since been developed and are in clinical use for ALK^+^ NSCLC [22, 23]. Mounting evidence suggests that patients with ALK-expressing malignancies eventually develop point mutations in ALK [14], rendering them insensitive to ALK inhibition and provoking reoccurrence of a more aggressive disease [6, 24]. Thus, there is a clear need for more effective therapies targeting alternative core signaling pathways.

We identified the AP-1 TFs, cJUN and JUNB, as downstream effectors of NPM-ALK [25], transcriptionally regulating the expression of Platelet Derived Growth Factor Receptor Beta *(Pdgfrb)* [26]. Strikingly, inhibition of PDGFRβ by the TKI imatinib (*Gleevec*) induced a complete and sustained remission in a late-stage relapsed ALK^+^ ALCL patient [26]. Furthermore, our findings resulted in the initiation of a clinical study evaluating imatinib efficacy according to PDGFR status [27]. Imatinib treatment can thus be considered as a promising therapeutic strategy for relapsed ALK^+^ ALCL. However, the mechanism of action and the extent of PDGFRβ involvement in ALCL pathogenesis still remains elusive.

In this study, we determine the influence of PDGFRβ on ALCL by developing a genetic ALK^+^ ALCL mouse model lacking PDGFRβ expression in neoplastic CD4^+^ T cells. Strikingly, genetic loss of *Pdgfrb* results in a significant increase in survival in line with the observed benefits of PDGFRβ kinase activity blockade via imatinib in patients. Mechanistically, we identify STAT5 as a novel downstream target of PDGFRβ in ALCL. Blockade of STAT5 results in a significant dampening of viability by mediating the pro-survival factor Bcl-x_L_ and the growth-enhancing cytokine IL-10. Additionally, we report the small molecule inhibitor AC-4-130 inhibits both STAT5 and STAT3 activation in our experimental setting, significantly inhibiting tumor development in vivo and highlighting the added benefit of a dual STAT3/5 inhibition. Overall, our findings suggest expression of PDGFRβ in ALCL stimulates a malignant boost, providing an attractive alternative/additive pathway for pharmacologic inhibition.

## Materials and methods

Additional materials and resources can be found in Suppl. Materials and Methods.

### Transgenic mouse strains

All animal experiments were performed in agreement with the ethical guidelines of the Medical University of Vienna and the Austrian Federal Ministry of Science and Research (Project number: BMWFW-66.009/0183-WF/V/3b/2017; BMWFW-66.009/ 0057-V/3b/2018; BMWFW-66.009/0401-V/3b/2018; 2020–0.103.412). Mice were kept in a pathogen-free environment under standard conditions. *Cd4*-NPM-ALK transgenic mice [28] were crossed with *Cd4*-Cre mice [29] and a second strain carrying loxP-flanked *Pdgfrb* (Exons 4–7) [30] in a *C57BL/6* x *BALB/c* mixed background resulting in the desired genotypes: *Cd4*-NPM-ALK^Tg/+^ *Cd4*-Cre^+/+^ Pdgfrb^fl/fl^ (*fl/fl Pdgfrb*) and *Cd4*-NPM-ALK^Tg/+^ *Cd4*-Cre^Tg/+^ Pdgfrb^Δ/Δ^ (*Δ/Δ Pdgfrb*) mice. Mice were genotyped using the primers listed in Suppl. Table 1.

### Human tissue microarrays

All human samples were obtained with informed written consent and in accordance with the Declaration of Helsinki.

#### Adult TMAs

Formalin-fixed paraffin-embedded (FFPE) tissue specimens from both male and female adult patients, diagnosed with systemic ALK^+^ (*n* = 25) or ALK^−^ (*n* = 25) ALCL, were obtained and reviewed by the local ethic boards of the Medical University of Vienna, Austria (no.1437/2016 and 1221/2019) and the University Hospital Brno, Czech Republic (no. 4–306/13/1). Diagnoses were assigned according to the WHO classification of lymphoid neoplasms.

#### Pediatric TMA

FFPE tissues specimens from 98 pediatric patients diagnosed with ALK^+^ ALCL included in the studies NHL-BFM90, NHL-BFM95 or enrolled in the European intergroup trial ALCL99 (NCT00006455) between 1992 and 2006, were obtained. All patients were treated with comparable Berlin-Frankfurt-Münster (BFM)-type chemotherapy, as previously described [12]. Eligibility was determined by detection of oncogenic NPM-ALK: either via NPM-ALK PCR, two color fluorescence in situ hybridization for the translocation t(2;5)(p23;35) or nuclear/cytoplasmic IHC staining for ALK. Staining procedures included bone marrow aspiration cytology and a spinal tap. Bone marrow involvement was defined by cytologically detectable ALCL cells, irrespective of cellular quantity. The studies were approved by the institutional ethics committee of the primary investigator of the NHL-BFM study group.

### Immunohistochemistry and whole tissue scans

IHC staining was performed with tissue fixed for 24 hours in formalin before embedding in paraffin blocks, as described [26]. Antibodies used for IHC can be found in Suppl. Table 2. A Panoramic MIDI slide scanner (3DHistech Ltd., Budapest, Hungary) with 40x optics was used to digitalize the tissue sections. Quantitation was performed using Definiens® TM Tissue Studio histomorphometry software (Definiens AG, Munich, Germany). Images were taken with a Zeiss AxioImager Z1 microscope.

### Multiplex immunobead cytokine assay

Murine *fl/fl Pdgfrb* and *Δ/Δ Pdgfrb* cells were cultured at a density 1 × 10^5^ cells in a 12-well plate and treated with AC-4-130 or DMSO as vehicle control. Post 72 hours of treatment, supernatants were collected and analyzed using ProcartaPlex antibody-based, magnetic bead reagent assay panels for multiplex protein quantitation using the Luminex xMAP technology and instrument platform. Concentrations of cytokines in the supernatants were determined simultaneously with the customized 27-plex immunoassay kit (ProcartaPlex Mouse 27-plex, ThermoFisher Scientific) using magnetic beads. Undiluted frozen samples (50 μL) were processed in 96-well plates according to the manufacturer’s instructions. Standard curves for each analyte were generated by measuring individual standards in duplicate and using the reference concentration supplied by the manufacturer. Measurement was performed on a calibrated Bio-Plex 200 system (Bio-Rad) in combination with Bio-Plex Manager software, version 6.1 (Bio-Rad). The concentrations were calculated from the standard curve using five-parameter logistic (5PL) regression curve fitting.

### ChIP-seq and initial processing

ChIP-seq was performed as previously described [31]. The antibodies used for each experiment are listed in Suppl. Table 3. For each ChIP, 5 μg of antibody coupled to 2 μg of magnetic Dynabeads (Life Technologies) was added to 3 ml of sonicated nuclear extract from formaldehyde-fixed cells. Chromatin was immunoprecipitated overnight, cross-links were reversed, and DNA was purified by precipitation with phenol:chloroform:isoamyl alcohol. DNA pellets were resuspended in 25 μl of TE buffer. Illumina sequencing, library construction, and ChIP-seq analysis methods were previously described [31]. Reads were aligned to the human reference genome (hg19) using bowtie v1.2.2 with parameters –k 2 –m 2 –best and –l set to the read length. For visualization, WIG files were created from aligned read positions using MACS v1.4 with parameters –w –S –space = 50 –nomodel –shiftsize = 200 to artificially extend reads to 200 bp and to calculate their density in 50-bp bins. Read counts in 50-bp bins were normalized to the millions of mapped reads, giving RPM values. WIG files were visualized in the IGV browser version 2.7.2.

### CUT&RUN sequencing and initial processing

CUT&RUN coupled with high-throughput DNA sequencing was performed using antibodies listed in Suppl. Table 3 and Cutana pA/G-MNase (EpiCypher) according to the manufacturer’s protocol. Briefly, cells were washed and incubated with activated concanavalin A beads for 10 min at room temperature. Cells were then resuspended in antibody buffer containing 0.01% digitonin, 1 ml of each antibody (Suppl. Table 3) was added to individual cell aliquots, and tubes were rotated at 4 °C overnight. The following day, targeted chromatin digestion and release were performed with 2.5 ml of Cutana pA/G-MNase and 100 mM CaCl_2_. Retrieved genomic DNA was purified with a MinElute PCR purification kit and eluted in 10 ml of buffer EB. Sequencing libraries were prepared with an automated Swift 2S system, followed by 100-bp paired-end sequencing with NovaSeq 6000. Reads were aligned to the human reference genome (hg19) using bowtie v1.2.2 in single-end mode with parameters –k 2 –m 2 –best and –l set to the read length. For visualization, WIG files were created from aligned read positions using MACS v1.4 with parameters –w –S –space = 50 –nomodel –shiftsize = 200 to artificially extend reads to 200 bp and to calculate their density in 50-bp bins. Read counts in 50-bp bins were then normalized to the millions of mapped reads, giving reads per million (RPM) values. WIG files were visualized in the Integrative Genomics Viewer (IGV) browser version 2.7.2. The antibodies used for CUT&RUN are listed in Suppl. Table 3.

### Data and code availability

Raw and processed data files were deposited to the NCBI GEO server. Code written in R/python to perform analysis of ChIP-seq and CUT&RUN are available upon request.

### Statistical analysis

Log-rank (Mantel-Cox) test, Student’s *t* test (Wilcoxon-Mann-Whitney correction), and half maximal inhibitory concentration (IC_50_) statistical analyzes were performed using GraphPad Prism® Software version 8. *P*-values were defined as indicated in the figure legends: ns = p > 0.05; * = p < 0.05; ** = p < 0.01; *** = p < 0.001; **** = p < 0.0001).

## Results

### PDGFRβ is a biomarker for aggressive ALK^+^ ALCL

**Fig. 1:**
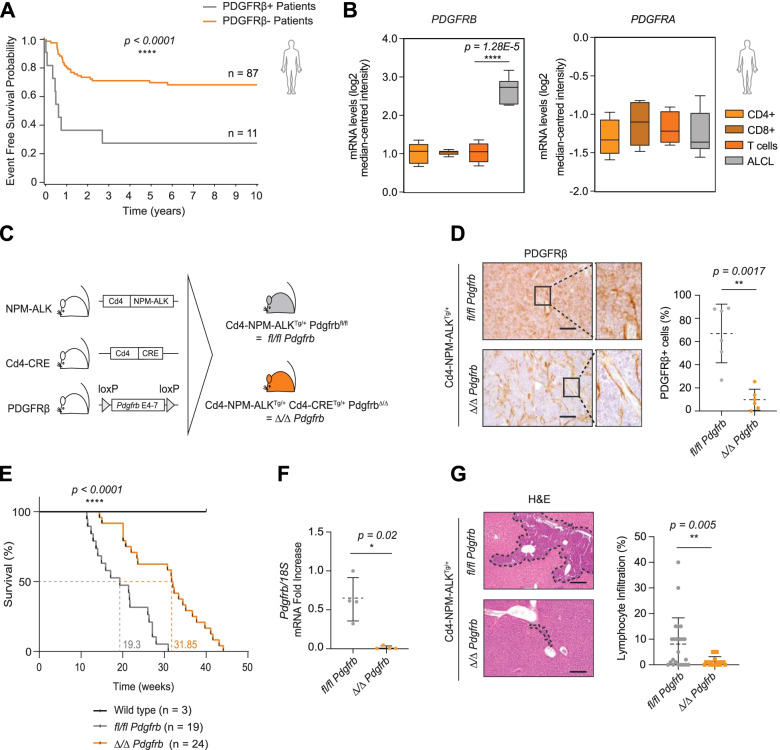
PDGFRβ is a biomarker for aggressive ALCL. **A** Tissue microarrays from 98 NPM-ALK^+^ ALCL patients enrolled to NHL-BFM 90 and 95 studies and the ALCL99 clinical trial, were stained for total PDGFRβ by immunohistochemistry (IHC) and expression levels were correlated with event-free survival (EFS). Staining was quantified according to high (*n* = 11; orange line) versus low (*n* = 87; grey line) PDGFRβ expression. **B** *PDGFRB* and *PDGFRA* mRNA transcript levels (log2 median-centered intensity) comparison between (*n* = 6) ALCL patients and (*n* = 10, T cells; *n* = 5, CD4^+^; and *n* = 5, CD8^+^) healthy donors of the Piccaluga dataset [32] extrapolated from the Oncomine database. **C** Schematic representation of the breeding strategy to obtain a genetic knockout of *Pdgfrb* in CD4^+^ T cells harboring the human NPM-ALK^+^ oncogenic fusion under the control of the *Cd4* enhancer. NPM-ALK^Tg^ littermates either expressing wild type *Pdgfrb* (*fl/fl Pdgfrb;* grey mouse) or lacking *Pdgfrb* in CD4^+^ T cells (***Δ****/****Δ*** *Pdgfrb;* orange mouse) were generated using Cre-mediated recombination driven by the *Cd4* promoter. **D** Representative pictures of PDGFRβ IHC analysis of *fl/fl Pdgfrb* and ***Δ****/****Δ*** *Pdgfrb* mouse thymomas at the experimental end point. Black squares in the left picture represent the area chosen for the magnification depicted on the right. Scale bars: 50 μm. Staining of (*n* = 6) end point thymic tumors per genotype were scanned and whole-slide quantification was performed using Definiens™ software. **E** Kaplan Meier cumulative survival analysis of (*n* = 3) wild type (black line), (*n* = 19) *fl/fl Pdgfrb* (grey line) and (*n* = 24) ***Δ****/****Δ*** *Pdgfrb* (orange line) mice in biological replicates. Values next to the dotted lines on the x-axis indicate median life expectancy for each genotype. **F** Quantitative RT-qPCR of *Pdgfrb* mRNA transcripts from (*n* = 4) *fl/fl Pdgfrb* (grey) and (*n* = 4) ***Δ****/****Δ*** *Pdgfrb* (orange) primary tumor cell lines. *Pdgfrb* levels were normalized to *18S* ribosomal RNA expression and depicted as fold-change over one *fl/fl Pdgfrb* biological replicate set to 1. **G** Hematoxilin and Eosin (H&E)-stained sections of tumor dissemination into the liver of two *fl/fl Pdgfrb* (grey) and ***Δ****/****Δ*** *Pdgfrb* (orange) mice at the experimental end point*.* The black dashed line represents malignant infiltration into secondary organs. Scale bars: 50 μm. **A** and **E** *p* values were determined by the log-rank (Mantel-Cox) test. **B**, **D**, **F** and **G** Data are shown as the means ± SD and *p* values were determined by the unpaired two-tailed Student’s t-test (ns = p > 0.05; * = p < 0.05; ** = p < 0.01; *** = p < 0.001; **** = p < 0.0001**)**

Imatinib treatment results in tumor regression in *Cd4*-NPM-ALK transgenic mice [26]. However, the mechanisms of action and whether this effect is dependent on NPM-ALK activity remained to be determined. This is particularly important as inhibition of alternative tumor-promoting pathways in combination with ALK inhibition might prevent the development of drug resistance [6]. We evaluated a set of ALCL biopsies, all taken from children treated with an identical BFM-based chemotherapy in three separate clinical trials, for PDGFRβ expression and correlated expression levels with clinical variables. Patients with PDGFRβ expression on tumor cells (*n* = 11) had a significantly lower five-year EFS compared to patients lacking (*n* = 87) membrane-bound PDGFRβ (Fig. 1A). We next analyzed an independent lymphoma patient dataset using the Oncomine database. In the six ALCL patient samples [32], we observed a significant upregulation of *PDGFRB* expression when compared to healthy donor CD4^+^ and CD8^+^ T cells. In contrast *PDGFRA* was not significantly upregulated (Fig. 1B). Having correlated PDGFRβ expression in patients with poorer EFS, we set out to map the molecular signatures activated by this receptor. We conditionally deleted *Pdgfrb* in CD4^+^ T cells in transgenic mice expressing the human NPM-ALK fusion oncogene under control of the murine *Cd4* promoter and enhancer (NPM-ALK^Tg^) [28], resulting in littermates either expressing wild type *Pdgfrb* (*fl/fl Pdgfrb*) or lacking *Pdgfrb* in CD4^+^ T cells (*Δ/Δ Pdgfrb*) (Fig. 1C). Regardless of sex, NPM-ALK^Tg^ mice, develop T cell lymphomas with a high penetrance [28]. We assessed and confirmed deletion of *Pdgfrb* in T cells by genotyping (Fig. S1A) and immunohistochemistry (Fig. 1D). NPM-ALK protein (Fig. S1B) and mRNA (Fig. S1C) were expressed at similar levels in *fl/fl Pdgfrb* and *Δ/Δ Pdgfrb* mice, suggesting that PDGFRβ does not influence NPM-ALK expression per se. Similarly, STAT3, an important downstream modulator of NPM-ALK, was strongly activated as demonstrated by tyrosine phosphorylation in 8 week-old thymi, confirming that NPM-ALK activity is independent of PDGFRβ expression (Fig. S1D) [33].

We next compared tumor development and survival rates of *fl/fl Pdgfrb* and *Δ/Δ Pdgfrb* mice. Strikingly, log-rank analysis of Kaplan-Meier survival curves indicated a 1.7-fold increase in survival for the *Pdgfrb* knockout mice (*fl/fl Pdgfrb* median survival: 19.3 weeks; *Δ/Δ Pdgfrb* median survival: 31.9 weeks) (Fig. 1E). Of note, both animal cohorts presented with identical thymic tumor phenotypes at the experimental end point (Fig. S1E), despite the total loss of *Pdgfrb* in tumor cells (Fig. 1F). These data suggest that PDGFRβ expression in tumor cells shortens the time to tumor development but does not prevent NPM-ALK driven lymphomagenesis. In addition to increased survival, *Δ/Δ Pdgfrb* mice had a decreased thymic tumor-to-body weight ratio (Fig. S1F), but spleen-to-body weight ratios were similar among the genotypes (Fig. S1G), consistent with an absence of splenic tumor development regardless of PDGFRβ status. Histopathological analysis of various organ parenchymas showed decreased dissemination of tumor cells to the kidney and liver in *Δ/Δ Pdgfrb* mice, whereas no significant differences were observed when comparing dissemination into the heart and lungs (Fig. 1G, S1H). Altogether, these results demonstrate that PDGFRβ expression facilitates ALK^+^ tumor formation and dissemination, and that our generated genetic mouse model is a valid tool to study the effects of PDGFRβ in ALK^+^ ALCL pathogenesis.

### PDGFRβ promotes autocrine growth of ALK^+^ ALCL cells via IL-10 secretion

**Fig. 2:**
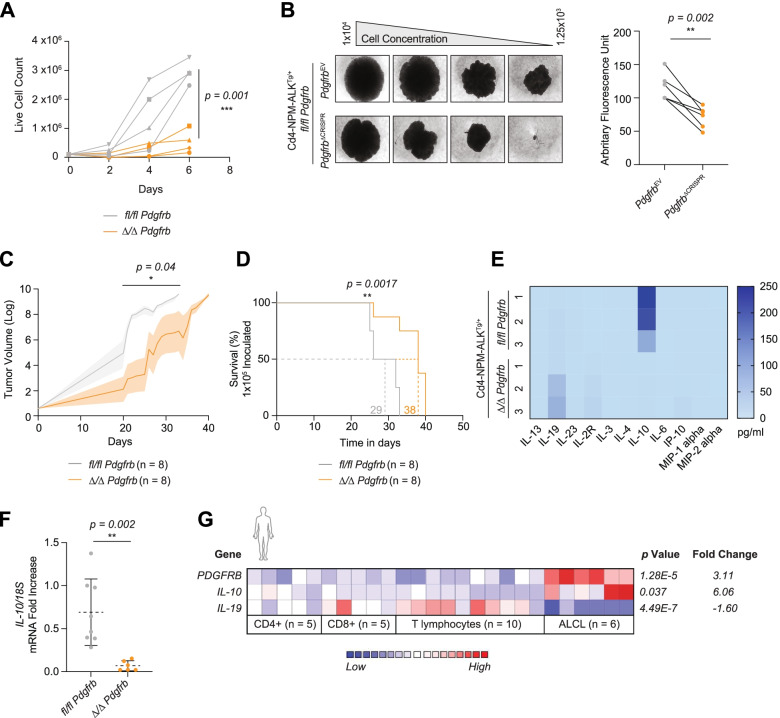
PDGFRβ promotes autocrine growth in ALCL. **A** Live cell counts of (*n* = 4) *fl/fl Pdgfrb* (grey) and (*n* = 4) *Δ/Δ Pdgfrb* (orange) primary mouse tumor cell lines seeded in 6-well plates at low confluency (1 × 10^5^ cells). Cells were stained with Trypan blue and live cell counts were recorded on days two, four and six. **B** Representative pictures of a *fl/fl Pdgfrb* cell line (*Pdgfrb*^*EV*^*;* empty vector transduced) subjected to CRISPR/Cas9 deletion of *Pdgfrb* (*Pdgfrb*^ΔCRISPR^) and seeded in a 96-well plate for a limiting dilution assay. Right graph: arbitrary fluorescence units measured using a resazurin assay were measured and correlated to the matched empty vector control (*n* = 5). **C** Longitudinal analysis of tumor volume increase of 6-week old immunodeficient (NOD.Cg-*Prkdc*^*scid*^, *Il2rd*^*tm1Wjl*^/SzJ) female mice inoculated with either (*n* = 8) *fl/fl Pdgfrb* (grey) or (*n* = 8) *Δ/Δ Pdgfrb* (orange) primary mouse tumor cell lines at a low concentration (1 × 10^5^ cells/flank). **D** Kaplan Meier cumulative survival analysis of 6-week old NSG female mice inoculated with either (*n* = 8) *fl/fl Pdgfrb* (grey) or (*n* = 8) *Δ/Δ Pdgfrb* (orange) primary mouse tumor cell lines at a low concentration (1 × 10^5^ cells/flank). Values next to the dotted lines on the x-axis indicate median life expectancy before tumor size reaches the 2000 mm^3^, the set threshold volume. **E** Heatmap depicting cytokine concentrations (pg/ml) in the supernatants of (*n* = 3) *fl/fl Pdgfrb* and (*n* = 3) *Δ/Δ Pdgfrb* primary mouse tumor cell lines cultivated in vitro at a low density (1 × 10^5^ cells). **F** Quantitative RT-qPCR of *Il-10* mRNA transcripts from (*n* = 8) *fl/fl Pdgfrb* (grey) and (*n* = 6) *Δ/Δ Pdgfrb* (orange) primary mouse tumor cell lines. Data was normalized to *18S* ribosomal RNA expression. **G** Gene comparison between (*n* = 6) ALCL patients and healthy donors of the Piccaluga dataset [32] extrapolated from the Oncomine database [34]. Heatmap colors represent z-scores normalized to depict relative values within rows, where blue represents least expressed and red represents most expressed. **A**, **B** and **F** Data are shown as means ± SD, and *p* values were determined by the unpaired two-tailed Student’s t-test. **D** *p* value was determined by log-rank (Mantel-Cox) test (ns = p > 0.05; * = p < 0.05; ** = p < 0.01; *** = p < 0.001; **** = p < 0.0001)

We next set out to address the mechanisms behind the observed prolongation in survival upon loss of PDGFRβ. Analysis of tumors at the experimental end point did not reveal any differences in the percentage of proliferating cells (Fig. S2A). Hence, we investigated proliferation capacity over time, as analysis of established tumors is a static observation and as such not representative of the process of lymphomagenesis. To do so, we performed in vitro proliferation assays seeding primary tumor cells at a low cell density (1 × 10^5^ cells). In this restricted growth environment, we observed decreased proliferation for *Δ/Δ Pdgfrb* primary tumor cells (Fig. 2A), consistent with delayed tumor development in *Δ/Δ Pdgfrb* mice observed in vivo (Fig. 1E). Comparable to primary tumor cell lines, CRISPR/Cas9 knock-out of PDGFRβ in ALK^+^ cells (Fig. S2B) also led to a decrease in proliferation when seeded at a low cell density (Fig. 2B). We next inoculated immunodeficient (NOD.Cg-*Prkdc*^*scid*^, *Il2rd*^*tm1Wjl*^/SzJ) mice with *fl/fl Pdgfrb* and *Δ/Δ Pdgfrb* primary tumor cells at low (1 × 10^5^) and ten-fold higher (1 × 10^6^) densities and monitored tumor initiation and development over time. Animals inoculated with a high density of *fl/fl Pdgfrb* cells reached the maximum tumor volume cut-off (2000 mm^3^) around 23 days post inoculation whereas animals inoculated with *Δ/Δ Pdgfrb* cells were sacrificed around 26 days post inoculation (Fig. S2C). We performed longitudinal analyzes of the tumor growth curves over the entire duration of the experiment and observed no difference in tumor volume between NSG mice inoculated with high density *fl/fl Pdgfrb* and *Δ/Δ Pdgfrb* cells (Fig. S2D). However, when NSG animals were inoculated with a low density of *Δ/Δ Pdgfrb* cells, we observed a significant delay in tumor growth (Fig. 2C) resulting in an average of 38 days to reach the experimental end point, compared to 29 days for the PDGFRβ expressing tumors (Fig. 2D).

Considering the role cytokines play in maintaining homeostatic T cell survival and proliferation, we hypothesized a change in the cytokine profile may be responsible for the observed effects. ProcartaPlex cytokine ELISA analysis indeed revealed that IL-10 was significantly reduced in the supernatant of *Δ/Δ Pdgfrb* tumor cells cultured in vitro (Fig. 2E, S2E), as were transcript levels of *IL-10* (Fig. 2F). In contrast, IL-19, a cytokine of the IL-10 super-family, was detected at higher levels in the supernatant of *Δ/Δ Pdgfrb* tumor cells, perhaps compensating for the lack of IL-10 (Fig. 2E, S2F). Interestingly, the six ALCL patients with upregulated *PDGFRB* expression [32] (Fig. 1B) also showed a downregulation of *IL-19* and an upregulation of *IL-10* mRNA levels in tumor cells compared to healthy control cells (Fig. 2G). Finally, using genome-wide DNA methylation data [35], we identified *IL-10* hypomethylation in five ALK^+^ ALCL patients when compared to peripheral blood-derived activated CD3^+^ T cells from five healthy donors (Fig. S2G). In contrast, human control cell lines showed DNA hypermethylation of the *IL-10* locus. In summary, our data suggests PDGFRβ orchestrates autocrine signaling in ALK^+^ ALCL cells that is sensitive to cellular density and IL-10 secretion.

### PDGFRβ activates STAT5 in ALK^+^ ALCL

**Fig. 3:**
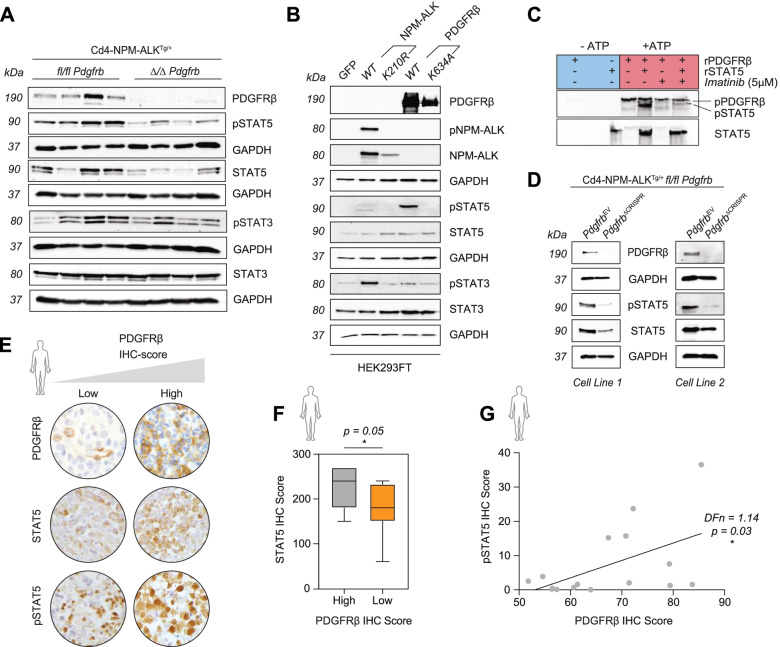
PDGFRβ activates STAT5. **A** Western blot showing protein levels of PDGFRβ, phospho (p) STAT3, total STAT3, phospho (p) STAT5 and total STAT5 in thymomas excised from (*n* = 4) *fl/fl Pdgfrb* and (*n* = 4) *Δ/Δ Pdgfrb* mice at the experimental end point. GAPDH serves as the loading control. The molecular weight of analyzed proteins in kiloDaltons (KDa) is shown on the left. **B** Western blot showing protein levels of PDGFRβ, phospho (p) NPM-ALK, total NPM-ALK, phospho (p) STAT5, total STAT5, phospho (p) STAT3 and total STAT3 in HEK293FT transfected cell lysates. GAPDH serves as the loading control. The molecular weight of analyzed proteins in kiloDaltons (KDa) is shown on the left. **C** Western blot showing phosphotyrosine-100 (pTyr-100) levels following a kinase assay with PDGFRβ and STAT5 recombinant (r) proteins. The kinase assay was performed in the absence (−) or presence (+) of ATP and 5 μM of Imatinib. The highlighted bands indicate phospho (p) PDGFRβ and (p) STAT5. Total STAT5 serves as the loading control. **D** Western blot showing protein levels of PDGFRβ, phospho (p) STAT5 and total STAT5 in two *fl/fl Pdgfrb* primary tumor cell lines subjected to CRISPR/Cas9 mediated deletion of *Pdgfrb*. GAPDH serves as the loading control. The molecular weight of analyzed proteins in kiloDaltons (KDa) is shown on the left. **E** Representative pictures of PDGFRβ, phospho-STAT5 and total STAT5 IHC analysis and grading scheme to quantify staining of tissue microarrays from human ALCL tumor samples. **F** PDGFRβ expression levels were divided into either high (*n* = 7; grey) or low (*n* = 13; orange) and correlated to total STAT5 as detected by IHC. **G** Correlation between phospho (p) STAT5 and total PDGFRβ IHC scores of ALCL tissue specimens (*n* = 16). Pearson correlation, *p =* 0.03 and *DFn =* 1.14. **F** data is shown as the mean ± SD, and the *p* value was determined by the unpaired two-tailed Student’s t-test (ns = p > 0.05; * = p < 0.05; ** = p < 0.01; *** = p < 0.001; **** = p < 0.0001)

It has recently been shown that resistance to ALK inhibition via crizotinib is mediated by aberrant upregulation of IL-10RA rewiring the STAT3 signaling pathway in ALCL. STAT3 consecutively binds to the promoters of *IL-10*, *IL-10RA* and *IL-10RB,* maintaining oncogenic signaling regardless of NPM-ALK phosphorylation [6]. As we detected elevated IL-10 levels in primary tumor cells expressing PDGFRβ, we hypothesized that overexpressed PDGFRβ might additionally fuel STAT3 activity in ALCL. Supporting this hypothesis, end stage tumors developing in *Δ/Δ Pdgfrb* animals indeed exhibited a decrease in both active STAT3 and STAT5 levels when compared to those from *fl/fl Pdgfrb* mice (Fig. 3A). Because both PDGFRβ and NPM-ALK are potent tyrosine kinases, we produced kinase dead (KD) versions of NPM-ALK (K210R or D309A) or PDGFRβ (K634A or D826A) via Site Directed Mutagenesis to further understand the phosphorylation cascade (Fig. S3A). PDGFRβ and NPM-ALK in either wild type (WT) or KD forms were expressed in HEK293FT cells and as expected, neither of the KD versions were phosphorylated on the indicated tyrosine residues normally associated with activity (Fig. S3B). As anticipated, WT NPM-ALK induced STAT3 phosphorylation [36] (Fig. 3B, S3C). In contrast, PDGFRβ selectively induced STAT5 phosphorylation (Fig. 3B, S3D), suggesting that NPM-ALK and PDGFRβ have preferential STAT3 or STAT5 tyrosine kinase substrates. This was confirmed by imatinib inhibition of PDGFRβ recombinant protein activity, which prevented STAT5 phosphorylation in an in vitro kinase assay (Fig. 3C). In addition, the *fl/fl Pdgfrb* cell lines subjected to CRISPR/Cas9 deletion of PDGFRβ similarly showed a dampening of phosphorylated and total STAT5 (Fig. 3D). Finally, co-staining for PDGFRβ and STAT5 indicated both factors are expressed in tumor cells and do not originate from different cell populations (Fig. S3E).

We next correlated PDGFRβ and STAT5 expression in ALCL patient biopsies by dividing samples into two cohorts according to PDGFRβ high and low IHC-scores (Fig. 3E). Interestingly, patients with a high PDGFRβ IHC-score also had high STAT5 expression levels (Fig. 3F). We analyzed a second cohort of 16 ALCL patient samples to validate our findings and found a significant positive correlation between PDGFRβ expression and active levels of STAT5 (Fig. 3G), supporting our in vitro findings.

### Inhibiting the PDGFRβ-STAT5 axis induces apoptosis of ALK^+^ ALCL cells

**Fig. 4:**
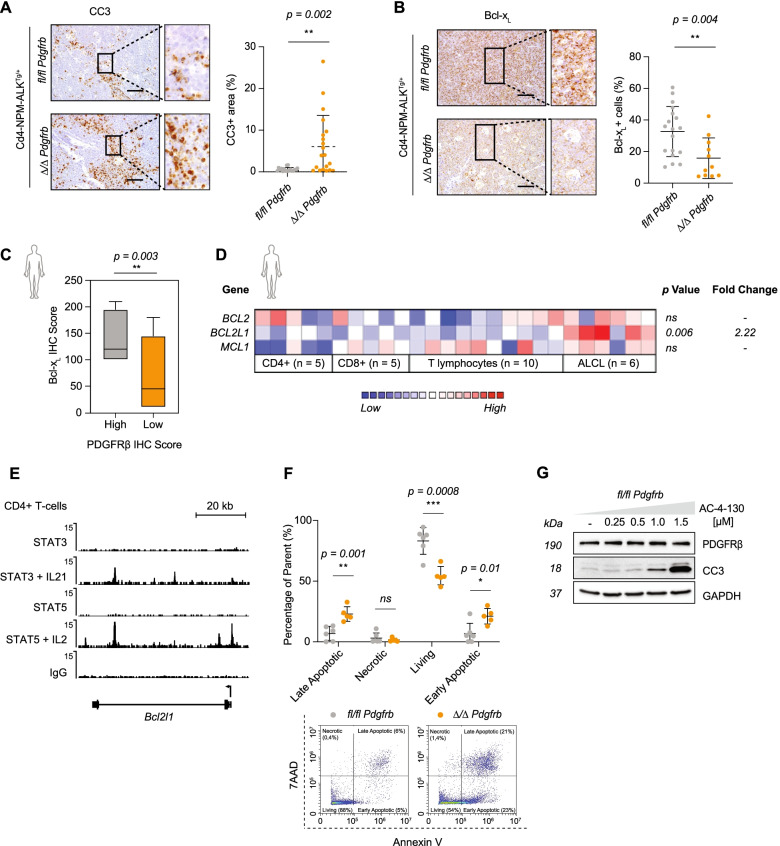
Inhibiting the PDGFRβ-STAT5 axis induces apoptosis. **A** Representative pictures of cleaved caspase 3 (CC3) IHC analysis of (*n* = 19) *fl/fl Pdgfrb* and (*n* = 20) *Δ/Δ Pdgfrb* mouse thymomas at the experimental end point. Black squares in the left pictures represent the area chosen for the magnification depicted on the right. Scale bars: 50 μm. Whole-slide scans were quantified using Definiens™ software (right graph). **B** Representative pictures of Bcl-x_L_ IHC analysis of (*n* = 18) *fl/fl Pdgfrb* and (*n* = 11) *Δ/Δ Pdgfrb* mouse thymomas at the experimental end point. Black squares in the left pictures represent the area chosen for the magnification depicted on the right. Scale bars: 50 μm. Whole-slide scans were quantified using Definiens™ software (right graph). **C** PDGFRβ expression levels were divided into either high (*n* = 7; grey) or low (*n* = 13; orange) and correlated to Bcl-x_L_ IHC of patient primary tumor samples. **D** *BCL2*, *MCL1*, *BCL2L1* (Bcl-x_L_) gene expression comparison between (*n* = 6) ALCL patients and healthy donors of the Piccaluga dataset [32] extrapolated from the Oncomine database [34]. Heatmap colors represent z-scores normalized to depict relative values within rows, where blue represents least expressed and red represents most expressed. **E** Normalized ChIP-seq alignment track for STAT3 and STAT5 (unstimulated or stimulated with either IL-2, or IL-21) in murine CD4+ T cells compared to IgG, shown at the gene loci for *Bcl2l1 (*Bcl-x_L_*).* Read densities (y-axis) were normalized to reads per million reads sequenced in each sample. **F** Representative FACS plots of *fl/fl Pdgfrb* and *Δ/Δ Pdgfrb* murine primary tumor cells co-stained with 7-aminoactinomycin D (7AAD) and Annexin V. The right graph indicates the percentage of necrotic, living, early or late apoptotic cells in both genotypes as indicated. **G** Western blot showing protein levels of CC3 and PDGFRβ in a *fl/fl Pdgfrb* primary tumor cell line following AC-4-130 treatment. GAPDH serves as the loading control. The molecular weight of analyzed proteins in kiloDaltons (KDa) is shown on the left. **A**, **B**, **C**, **D** and **E** Data are shown as means ± SD, and *p* values were determined by the unpaired two-tailed Student’s t-test (ns = p > 0.05; * = p < 0.05; ** = p < 0.01; *** = p < 0.001; **** = p < 0.0001)

STAT5 plays an essential role in maintaining self-renewal capacities of hematopoietic cells via the tight regulation of downstream targets such as *Granzyme B*, *Perforin*, *Osm*, *Hif2a*, and *Bcl-2* family members [37, 38]. As STAT5 has been reported to have anti-apoptotic functions in hematopoietic cells [39], we next assessed apoptosis in the murine tumors. IHC analysis for Cleaved Caspase 3 (CC3) revealed an increase in clusters of apoptotic cells in *Δ/Δ Pdgfrb* tumors compared to *fl/fl Pdgfrb* controls (Fig. 4A). This increase in apoptotic clusters correlated with a decrease in levels of STAT5 (Fig. S4A) and its anti-apoptotic target Bcl-x_L_ (Fig. 4B). The ALCL patient biopsies, previously identified to have high PDGFRβ IHC-scores, also had elevated Bcl-x_L_ levels (Fig. 4C, S4B), indicating that our murine model mirrors the molecular signatures observed in human patients.

To confirm the importance of Bcl-x_L_ in ALCL, we made use of the Piccaluga Lymphoma dataset [32] and observed significant upregulation of *BCL2L1* (Bcl-x_L_) expression when compared to healthy donor T cells, whereas *BCL2* and *MCL1* were not significantly upregulated (Fig. 4D). These data suggest that STAT5 drives cell survival by activation of pro-survival pathways preventing apoptosis. Indeed, by re-analyzing several publicly available ChIP-Seq datasets derived from murine and human cell lines or ex-vivo models, we identified binding of both active STAT3 and STAT5 to the promoter of Bcl-x_L_ (Fig. 4E, S4C and D). As a biological read-out, we performed a 7AAD/Annexin V FACs co-staining and identified higher levels of apoptosis in our primary tumor cells (Fig. 4F, S4E), whereas the cell cycle was not significantly affected (Fig. S4F). Western blot analysis revealed that inhibition of STAT activity using the SH2 domain small molecular weight inhibitor AC-4-130, successfully induces apoptosis via CC3 (Fig. 4G, S4G). Our data suggest that genetic deletion or inhibition of the PDGFRβ-STAT5 axis induces a CC3-mediated apoptotic phenotype in ALK^+^ ALCL.

### STAT5A and STAT5B are essential for unrestricted cell proliferation

**Fig. 5:**
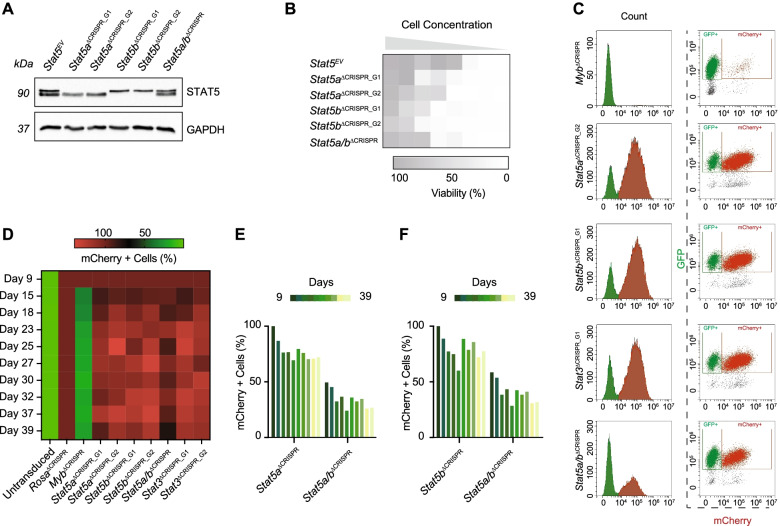
STAT5A/B are essential for unrestricted proliferation. **A** Western blot showing protein levels of phospho (p) STAT5 and total STAT5 in *fl/fl Pdgfrb* following CRSIPR/Cas9 mediated knockout of either STAT5A, STAT5B or both genes. GAPDH serves as the loading control. The molecular weight of analyzed proteins in kiloDaltons (KDa) is shown on the left. **B** Heatmap depicting cell viability of *fl/fl Pdgfrb* primary tumor cells following CRSIPR/Cas9 deletion of either STAT5A, STAT5B or both genes. Cells were seeded in a 96-well plate in limiting dilutions and arbitrary fluorescence units were measured using a resazurin assay. **C** Representative FACS plots of *Stat5a*^*Δ*CRISPR^, *Stat5b*^*Δ*CRISPR^, *Stat5a/b*^*Δ*CRISPR^, *Stat3*^*Δ*CRISPR^ or *Myb*^*Δ*CRISPR^ cells 39 days post-transduction. The left graphs represent ‘Count vs. mCherry’ and the right graphs represent ‘GFP (FITC-A channel) vs. mCherry (ECD-A channel)’. **D** Heatmap representing the survival of Lenti-EF1As-Cas9-P2A-GFP and U6-IT-mPgk-mCherry vector expressing cells over time. Viability was calculated as the percentage of mCherry+ cells relative to the negative non-targeting control (*Rosa*^*Δ*CRISPR^) for each condition on day 9. *Myb*^*Δ*CRISPR^ was used as a positive control. **E** Cell viability of *Stat5a/b*^*Δ*CRISPR^ double knock out cells normalized to individual *Stat5a*^*Δ*CRISPR^ cells over time. Data is plotted as the percentage of mCherry+ cells relative to Day 9 post-transduction. **F** Cell viability of *Stat5a/b*^*Δ*CRISPR^ double knock out cells normalized to individual *Stat5b*^*Δ*CRISPR^ cells over time. Data is plotted as the percentage of mCherry+ cells relative to Day 9 post-transduction. **B**, **D**, **E** and **F** Data are shown as means ± SD

To determine whether the two STAT5 gene products mediate apoptosis to the same extent on a PDGFRβ+ background, we proceeded to delete either STAT5A, STAT5B or both genes simultaneously (Fig. S5A). CRISPR/Cas9 deletion of either *Stat5a* or *Stat5b* resulted in complete gene knock-out within the bulk population. On the contrary, the guide RNA designed to target both *Stat5a*/*b* only resulted in a knockdown of either gene product within the bulk population (Fig. 5A). Knockdown of *Stat5a*/*b* resulted in a decrease in proliferation similar to that achieved following single *Stat5a* or *Stat5b* knockout (Fig. 5B), suggesting at least one of the two gene products is necessary for survival. To confirm this hypothesis, we next attempted to isolate single clones from *Stat5a/b*^*Δ*CRISPR^ bulk populations. Sanger sequencing coupled with Western blot analysis suggested simultaneous deletion of *Stat5a*/*b* is lethal for ALK^+^ ALCL cells as we never obtained a clone with a complete deletion (Fig. S5B).

To track the effects of the double deletion of *Stat5a*/*b* over time we next performed a CRISPR/Cas9-based competition assay (Fig. S5C). We firstly transduced our primary tumor cell line derived from a *fl/fl Pdgfrb* mouse with a vector expressing *SpCas9* and GFP. Following validation, we transduced the stably *SpCas9*-GFP-expressing clones with a vector expressing our sgRNAs of interest and mCherry. A competition assay was used to monitor competing growth kinetics of sgRNA-expressing mCherry and *SpCas9*-GFP positive (mCherry+) cells versus non-targeting *SpCas9*-GFP (GFP+) cells. Deletion of either *Stat5a*, *Stat5b* or *Stat3* did not result in a significant decrease in viability indicated by the elevated expression of mCherry+ cells 39 days post transduction (Fig. 5C, S5D). On the contrary, the double deletion of *Stat5a*/*b* resulted in a decrease in mCherry+ vs GFP+ cells, suggesting the loss of both gene products impacts viability (Fig. 5D). Normalizing the percentage of *Stat5a/b*^*Δ*CRISPR^ mCherry+ cells to either *Stat5a*^*Δ*CRISPR^ or *Stat5b*^*Δ*CRISPR^ indicated no selective preference for either gene product over time (Fig. 5E, F). These data suggests that loss of *Stat5a*/*b* is lethal for PDGFRβ^+^ ALK^+^ ALCL cells, providing a rational for the use of STAT inhibitors.

### Targeting STAT3/5 in vivo is therapeutically relevant in ALK^+^ ALCL

**Fig. 6:**
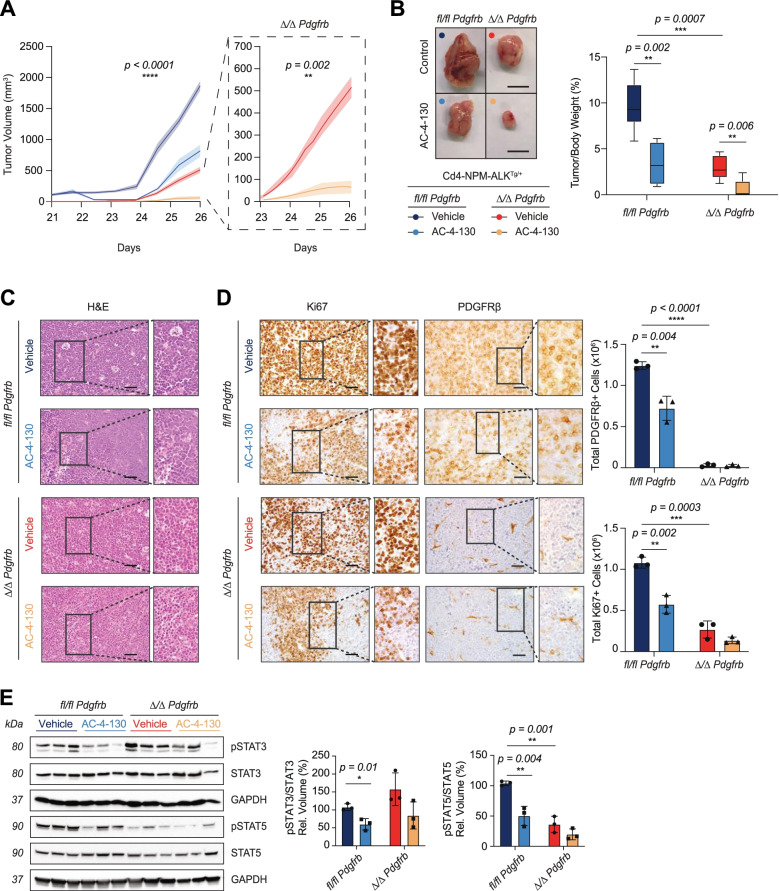
Targeting STAT5 in vivo is therapeutically relevant for ALK^+^ ALCL. **A** The tumor volume (mm^3^) increase of (*n* = 3) *fl/fl Pdgfrb* and (*n* = 3) *Δ/Δ Pdgfrb* inoculated 6-week old female NSG mice treated with either vehicle or AC-4-130. **B** Representative macroscopic pictures of excised left and right flank tumors. The right graph represents tumor weight to body weight ratio (%) at the experimental end point. **C** Representative pictures of the H&E analysis of (*n* = 3) *fl/fl Pdgfrb* and (*n* = 3) *Δ/Δ Pdgfrb* inoculated 6-week old female NSG mice treated with either vehicle or AC-4-130. The black squares in the left pictures represent the area chosen for the magnification depicted on the right. Scale bars: 50 μm. **D** Representative pictures of Ki67 and PDGFRβ IHC analysis of (*n* = 3) *fl/fl Pdgfrb* and (*n* = 3) *Δ/Δ Pdgfrb* inoculated 6-week old female NSG mice treated with either vehicle or AC-4-130. The black squares in the left pictures represent the area chosen for the magnification depicted on the right. Scale bars: 50 μm. Whole-slide scans were quantified using Definiens software (right graphs). **E** Western blot showing protein levels of phospho (p) STAT3, total STAT3, phospho (p) STAT5 and total STAT5 of end point tumors excised from (*n* = 3) *fl/fl Pdgfrb* and (*n* = 3) *Δ/Δ Pdgfrb* inoculated 6-week old female NSG mice treated with either vehicle or AC-4-130. GAPDH serves as the loading control. The molecular weight of analyzed proteins in kiloDaltons (KDa) is shown on the left. Phospho (p) STAT3 levels over total STAT3 and phospho (p) STAT5 levels over total STAT5 are depicted as relative volume in %. **B**, **D**, and **E** Data are shown as means ± SD, and *p* values were determined by an unpaired two-tailed Student’s t-test (multiple t-test application on GraphPad) (ns = p > 0.05; * = p < 0.05; ** = p < 0.01; *** = p < 0.001; **** = p < 0.0001)

Having identified the PDGFRβ-STAT5 axis as an important oncogenic driver in ALCL, we next assessed the efficacy of the STAT inhibitor AC-4-130 in vivo. The original publication that identified AC-4-130 as a selective STAT5 inhibitor, postulated AC-4-130 might also influence STAT1 and STAT3 activity [40]. Due to the dependance of the JAK/STAT signaling pathway in ALCL we hypothesized AC-4-140 would be an attractive therapeutic strategy in our PDGFRβ+ ALCL model. We engrafted *fl/fl Pdgfrb* and *Δ/Δ Pdgfrb* tumor cells subcutaneously into 6-week old NSG mice. Once tumors reached palpable dimensions, mice were treated daily with either vehicle (10% DMSO, 5% Cremophore in PBS) or single agent AC-4-130 (25 mg/kg) (Fig. S6A). AC-4-130 treatment administered via intraperitoneal injection was well tolerated with no significant decrease in animal weight nor toxicity (Fig. S6B, C and [40]). Longitudinal analysis of tumor volume over time indicated that *Δ/Δ Pdgfrb* tumors grew at a significantly slower rate than *fl/fl Pdgfrb* tumors, mirroring the phenotype observed in the transgenic mouse model (Fig. 1E). Strikingly, single agent AC-4-130 treatment led to a delay in tumor growth in both experimental cohorts relative to vehicle controls (Fig. 6A). All experimental animals were sacrificed when the *fl/fl Pdgfrb* vehicle control tumors reached 2000 mm^3^ in size. At the experimental end point, AC-4-130 treated mice, in both cohorts, showed a significant decrease in tumor-to-body weight ratio (Fig. 6B). This experiment was repeated with a second set (biological replicates) of *fl/fl Pdgfrb* and *Δ/Δ Pdgfrb* tumor cells. Consistently, treatment with AC-4-130 resulted in a significant decrease of tumor volume (Fig. S6D) and in tumor-to-body weight ratio (Fig. S6E). Thus, our data suggest that STAT3/5 inhibition is a rational therapeutical option for ALK^+^ ALCL irrespective of PDGFRβ expression status.

Residual masses following AC-4-130 treatment were harvested and morphologically analyzed via IHC. Macroscopically, xenografted tumors recapitulated the histological features of ALK^+^ tumors derived from *Cd4*-NPM-ALK transgenic mice. H&E staining revealed small, round monomorphic cells with characteristically necrotic/apoptotic regions in the treated tumors (Fig. 6C). Consistent with treatment efficacy, immunostaining for Ki67 revealed a significant decrease in proliferating lymphoma cells upon AC-4-130 treatment (Fig. 6D). As previously reported, AC-4-130 treatment also resulted in a decrease in PDGFRβ expression (Fig. 6D and [40]). Western blot analysis of excised tumors indicated that AC-4-130 treatment decreases the activity of STAT5 and STAT3 in vivo (Fig. 6E and S6F), potentially due to the interwoven signaling network between NPM-ALK/STAT3 and PDGFRβ/STAT5.

## Discussion

Imatinib has previously been identified as an effective treatment for PDGFRβ^+^ ALK^+^ ALCL relapse patients, however the mechanisms of action and the factual contribution of PDGFRβ remained unanswered [26]. We show here that T cell-specific deletion of PDGFRβ in a genetically engineered mouse model mimicking human ALCL in vivo, leads to delayed tumor growth and prolonged survival. To decipher the central signaling executor amongst the large repertoire of downstream targets activated by PDGFRβ, we used a tool kit of in vitro and in vivo models and identified that PDGFRβ signals preferentially via STAT5. The activation of the PDGFRβ/STAT5 axis in tumor cells results in a malignant boost by increasing autocrine-dependent proliferation and evading apoptosis. Treatment with the STAT5 SH2-domain inhibitor AC-4-130, results in a reduction in tumor growth in ALK^+^ ALCL, irrespective of PDGFRβ expression. Interestingly, we observed a decrease in both STAT3 and STAT5 following in vivo AC-4-130 treatment. This effect on STAT3 and STAT5 was already observed by Wingelhofer et al.*,* who discussed that AC-4-130 targets first cellular STAT5 at pharmacologically relevant concentrations while having smaller effects on STAT3 and STAT1 [40]. We therefore suggest these “smaller effects” are accentuated in diseases such as ALCL that centrally rely on STAT3 activity to maintain malignancy. Our findings suggest that STAT5 plays a crucial role in ALCL oncogenesis and that inhibition of the PDGFRβ/STAT5 axis, but also of the NPM-ALK-STAT3 axis, both upstream via imatinib/ALK inhibitors and downstream with AC-4-130, is therapeutically relevant in ALK^+^ ALCL.

Murine thymic lymphoma cells lacking PDGFRβ proliferate at slower rates both in vitro (Fig. 2A) and when implanted subcutaneously into NSG mice (Fig. 2C)*.* In contrast to ALK^+^ T cells lacking *Tyk2* [8], deletion of PDGFRβ resulted in reduced proliferation suggesting that PDGFRβ acts as a malignant boost. Cytokine screening indicated that IL-10 is significantly downregulated in *Δ/Δ Pdgfrb* primary tumor cell lines providing an explanation for the observed density-dependent proliferation defect. IL-10 is one of the most abundant cytokines in peripheral blood of children diagnosed with ALCL [41] and it is readily detected in both systemic and cutaneous ALCL [42]. Our data echoes previous findings which demonstrated that IL-10 is central for TYK2-mediated STAT1 and STAT3 activity in ALCL [8]. More recently, the IL-10R was found to directly activate STAT3, bypassing NPM-ALK, to bind to the promoters of *IL-10, IL-10RA* and *IL-10RB* [6]. Our findings, which are supported by two independent lymphoma patient datasets (Fig. 1B), propose PDGFRβ is yet another potent kinase in the list of regulators of IL-10 expression in ALCL and that targeting the PDGFRβ/STAT5/IL-10 axis is an attractive therapeutic strategy. In parallel to the observed decrease of IL-10, we detected an increase in secreted IL-19 upon loss of PDGFRβ (Fig. 2E). IL-19 is a member of the IL-10 cytokine superfamily and forms part of a gene cluster alongside *IL-10* and *IL-20* located on chromosome *1q32* [43, 44]. Interestingly IL-19, which has been reported to play both pro- and anti-inflammatory roles, induces STAT1 and STAT3 activation via binding to IL-20Rα and IL20-Rβ [44, 45]. It is thus attractive to speculate that in the absence of PDGFRβ, alternative pathways capable of mediating STAT phosphorylation, such as IL-19/IL-20Rα/β-STAT3 node, could compensate for decreased STAT activity.

To date STAT3 [46] dominates the spotlight of ALCL research although recent findings have begun to unravel the important involvement of STAT1 [8] and STAT5 [47]. In particular, STAT5A/B exhibit important functions in the pathogenesis of hematological neoplasias due to their involvement in lymphoid and myeloid cell differentiation [38, 48–50]. In human ALK^+^ ALCL cell lines, the two different STAT5 gene products were identified to play divergent functions [9]. Nevertheless, our data indicate both STAT5A and STAT5B are equally expressed in PDGFRβ^+^ ALK^+^ ALCL. Deletion of either *Stat3*, *Stat5a* or *Stat5b* did not result in cell death nor a detectable upregulation of other *Stat* family members, advocating for a defined *Stat* function during ALK^+^ ALCL development. However, we did not achieve a complete knockout of both *Stat5a*/*b* gene products simultaneously, suggesting that at least one of these remained active and was sufficient to maintain cellular homeostasis (Fig. 5D, S5B). In contrast to peripheral T cell leukemia/lymphoma (PTCL) [48, 51, 52], we did not observe a selective preference for maintenance of either *Stat5a* or *Stat5b* in malignant ALK^+^ cells expressing PDGFRβ. Kinetic tracking of viability, using mCherry expression, indicated knockdown of both *Stat5a/b* resulted in a significant decrease in cell viability, however this population was not completely lost over time, hinting towards sequential activation of STAT3/5 activity in ALK^+^ ALCL.

The elevated levels of STAT3/5 in ALK^+^ patient samples, renders them attractive therapeutic targets as blocking would preferentially affect malignant lymphocytes while leaving homeostatic lymphopoiesis unaffected. In vivo and in vitro AC-4-130 treatments resulted in an induction of apoptosis mediated via CC3 and Bcl-x_L_ in ALK^+^ lymphocytes. Interestingly, we found that STAT3 binds to super-enhancers that regulate high expression of Bcl-x_L_ in both ALK^+^ and ALK^−^ ALCL lymphoma cell lines [53] (Fig. S4C). Further in silico investigations revealed binding of STAT3 and STAT5 in murine primary CD4+ T cells and hematopoietic precursor cells (HPC7) [54, 55], suggesting a potential co-dependence at a transcriptional level (Fig. 4E, S4D). We postulate that this mode of action might be maintained in other tumors expressing elevated PDGFRβ levels such as cervical cancer [56] and castration-resistant prostrate cancer [57]. Interestingly, STAT3 and STAT5 hyperactivation has also been reported in these models [58, 59], rendering them potential druggable PDGFRβ targets. Extrapolating our findings from ALCL into a more general context, we hypothesize that multi-layered combinatorial treatments targeting the PDGFRβ-STAT5 signaling axis via imatinib upstream and AC-4-130 downstream might bolster response rates. Targeting single or multiple pathways at different hierarchical levels would in theory reduce the possibility of developing resistance to one specific drug. Nevertheless, future investigations into STAT small molecule inhibitors and degraders are urgently required to better understand the off-target effects.

In conclusion, we identify the PDGFRβ/STAT5 axis acts as a booster of malignancy, operating in parallel to the oncogenic NPM-ALK-STAT3 signaling cascade, resulting in a more aggressive ALCL disease entity. Our results highlight the importance of both STAT5A and STAT5B in ALK^+^ ALCL and suggest that blockade of STAT3/5 activity represents a new therapeutic strategy for the treatment of ALCL refractory patients. However, the use of STAT inhibitors will need to be rigorously assessed for their clinical applicability in the future. Thus, targeting activators upstream of the STATs, such as PDGFRβ and NPM-ALK, remains a highly relevant approach.

## Supplementary Information

**Additional file 1: Supplementary Fig. 1. A).** Genotyping PCR from purified genomic mouse tail DNA after *Cre* expression in *fl/fl Pdgfrb* and *Δ/Δ Pdgfrb* samples. Depicted are results for *ALK*, *Cd4*-*CRE* and the *Pdgfrb* alleles (without *loxP* sites: wild type (wt); with *loxP* sites: *fl*/*fl*) and the recombined (∆) *Pdgfrb* locus. Genomic mouse tail DNA from a PDGFRβ fl/+ animal was used as the PDGFRβ genotyping PCR positive control. Expected fragment band sizes: *ALK* (TG/+: 177 bp), *Cd4*-*CRE* (TG/+: 316 bp) and the *Pdgfrb* (WT: 498 bp and fl/fl: 562 bp). **B).** Representative pictures of ALK IHC analysis of *fl/fl Pdgfrb* and *Δ/Δ Pdgfrb* mouse thymomas at experimental end point. Scale bars: 50 μm. Black squares in the left picture represent the area chosen for the magnification depicted on the right. Stainings of (*n* = 9) tumor samples per genotype were scanned and whole-slide quantified using the Definiens™ software. **C).** Quantitative RT-qPCR based quantification of *NPM-ALK* mRNA transcripts from: (*n* = 4) control animals (black); (*n* = 4) *Cd4*-CRE^*Δ/Δ Pdgfrb*^ lacking NPM-ALK oncogene (pink); (*n* = 7) *fl/fl Pdgfrb* 8 week-old developing thymi (dark grey); (*n* = 6) *Δ/Δ Pdgfrb* 8 week-old developing thymi (red); (*n* = 5) end point thymomas (light grey); and (*n* = 8) end point thymomas (orange). Data was normalized to *18S* ribosomal RNA and depicted as a fold-change over one *fl/fl Pdgfrb* biological replicate set to 1. **D).** Western blot analysis showing protein levels of phospho (p) STAT3 and total STAT3 for 8 week-old pre-thymoma lysates: (*n* = 3) control, (*n* = 3) *fl/fl Pdgfrb* and (*n* = 3) *Δ/Δ Pdgfrb*. GAPDH serves as loading control. The molecular weight of analyzed proteins in kiloDaltons (KDa) is shown on the left. **E).** Left panel: representative macroscopic pictures of thymomas of *fl/fl Pdgfrb* and *Δ/Δ Pdgfrb* mice resected at experimental end point. Scale bar: 3 cm. Right panels: representative H&E-stained sections of tumors from *fl/fl Pdgfrb* and *Δ/Δ Pdgfrb* mice at experimental end point*.* Black squares in the middle picture represent the area chosen for the magnification depicted on the right. Scale bars: 50 μm. **F).** Thymic tumor weight from (*n* = 17) *fl/fl Pdgfrb* (grey) and (*n* = 17) *Δ/Δ Pdgfrb* (orange) mice normalized to whole body weight at experimental end point. **G).** Spleen weight from (*n* = 17) *fl/fl Pdgfrb* (grey) and (*n* = 17) *Δ/Δ Pdgfrb* (orange) mice normalized to whole body weight at experimental end point. **H).** H&E-stained sections of tumor dissemination into the kidney, heart and lungs from *fl/fl Pdgfrb* and *Δ/Δ Pdgfrb* mice at experimental end point*.* Black dashed line represents malignant infiltration into secondary organ. Scale bars: 50 μm. Individual biological replicates used for statistical analyzes are shown in the graph below. **B, C, F, G** and **H** Data are shown as mean ± SD, and *p* values were determined by unpaired two-tailed Student’s t-tests (ns = p > 0.05; * = p < 0.05; ** = p < 0.01; *** = p < 0.001; **** = p < 0.0001). **Supplementary Fig. 2. A).** Representative pictures of Ki67 IHC analysis of (*n* = 9) *fl/fl Pdgfrb* and (*n* = 7) *Δ/Δ Pdgfrb* murine thymomas at experimental end point. Black squares in the left pictures represent the area chosen for the magnification depicted on the right. Scale bars: 50 μm. Whole-slide scans were quantified using the Definiens software. **B).** Schematic representation of three individual CRISPR guide RNAs designed to target the genomic murine *Pdgfrb* locus. The sequence of the guide RNA is depicted in blue and the protospacer adjacent motif (PAM) in red. Right panel: Western blot showing protein levels of PDGFRβ in three *fl/fl Pdgfrb* primary tumor cell lines subjected to CRISPR/Cas9 deletion of *Pdgfrb* (*Pdgfrb*^ΔCRISPR^) and empty vector transduced control (*Pdgfrb*^*EV*^*)*. GAPDH serves as loading control. The molecular weight of analyzed proteins in kiloDaltons (KDa) is shown on the left. **C).** Kaplan Meier cumulative survival analysis of 6 week-old NSG females inoculated with either (*n* = 8) *fl/fl Pdgfrb* (grey) or (*n* = 8) *Δ/Δ Pdgfrb* (orange) primary mouse tumor cell lines at a high concentration (1 × 10^6^ cells/flank). Values next to the dotted lines on the x-axis indicate median life expectancy before tumor size reaches the 2000 mm^3^ set threshold volume. **D).** Longitudinal analysis of tumor volume increase of 6 week-old NSG female mice inoculated with either (*n* = 8) *fl/fl Pdgfrb* (grey) or (*n* = 8) *Δ/Δ Pdgfrb* (orange) primary mouse tumor cell lines at a high concentration (1 × 10^6^ cells/ flank). **E).** Cytokine assay measuring IL-10 concentration (pg/ml) of (*n* = 3) *fl/fl Pdgfrb* (grey) and (*n* = 3) *Δ/Δ Pdgfrb* (orange) primary mouse tumor cell lines. **F).** Bar chart depicting IL-10 (red) and IL-19 (blue) concentration (pg/ml) in the supernatant of (*n* = 3) *fl/fl Pdgfrb* and (*n* = 3) *Δ/Δ Pdgfrb* primary mouse tumor cell lines seeded at a low density (1 × 10^5^). **G).** Differential genomic DNA methylation on the human *IL-10* locus. Top panel: ALK^+^ (*n* = 5) and ALK^−^ (*n* = 5) ALCL patient samples compared to (*n* = 5) healthy CD3^+^ T cells. Data were retrieved from Hassler et al.*,* 2016. Middle panel: UCSC gene annotation track indicating *IL-10* gene. Lower panel: CpG Methylation obtained from Methyl 450 K Bead Arrays from ENCODE/HAIB depicting HL-60, Jurkat, K562, T-47D and H1-hESC human cell lines. Orange: methylated (score > = 600), Purple: partially methylated (200 < score < 600), Bright Blue: unmethylated (0 < score < = 200). **A** and **E** Data are shown as mean ± SD and *p* values were determined by unpaired two-tailed Student’s t-tests. **C** *p* value was determined by log-rank (Mantel-Cox) test (ns = p > 0.05; * = p < 0.05; ** = p < 0.01; *** = p < 0.001; **** = p < 0.0001). **Supplementary Fig. 3. A).** Schematic representation of the wild type and mutated kinase dead versions of either NPM-ALK (green) or PDGFRβ (red). Exons depicted in grey, altered amino acids in blue and hashtag represents mutated nucleotide. **B).** Western blot showing phosphotyrosine-100 (pTyr-100) levels in HEK293FT cells transfected with either wild type or kinase dead versions of PDGFRβ or NPM-ALK. **C).** Western blot analysis showing protein levels of phospho (p) NPM-ALK, total NPM-ALK, phospho (p) STAT3 and total STAT3 in HEK293FT transfected cell lysates. GAPDH serves as loading control. The molecular weight of analyzed proteins in kiloDaltons (KDa) is indicated. **D).** Western blot showing protein levels of PDGFRβ, phospho (p) STAT5 and total STAT5 in HEK293FT transfected cell lysates. GAPDH serves as loading control. The molecular weight of analyzed proteins in kiloDaltons (KDa) is shown on the left. Phospho (p) STAT5 levels over total STAT5 are depicted as relative volume in %. **E).** Double immunofluorescence staining of *fl/fl Pdgfrb* and *Δ/Δ Pdgfrb* primary tumor cells derived from our transgenic Cd4-NPM-ALKTg/+ model. Cells were fixed and stained with antibodies against PDGFRβ (green) and STAT5 (red). Cells were counterstained with DAPI (blue). Pictures were acquired with identical pixel density, image resolution, and exposure time using a confocal LSM Observer Z.1 Zeiss Microscope. Scale bars: 5 μm. **C** and **D** Data are shown as mean ± SD and *p* values were determined by unpaired two-tailed Student’s t-tests (ns = p > 0.05; * = p < 0.05; ** = p < 0.01; *** = p < 0.001; **** = p < 0.0001). **Supplementary Fig. 4. A).** Representative pictures of total STAT5 IHC analysis of (*n* = 8) *fl/fl Pdgfrb* and (*n* = 7) *Δ/Δ Pdgfrb* mouse thymomas at experimental end point. Black squares in the left pictures represent the area chosen for the magnification depicted on the right. Scale bars: 50 μm. Whole-slide scans were quantified using the Definiens™ software (right graph). **B).** Representative pictures of Bcl-x_L_ IHC analysis and the PDGFRβ grading scheme used to quantify staining of tissue microarrays from human ALCL tumor samples. **C).** Normalized ChIP-seq alignment track for STAT3 in MAC1 cells, and normalized CUT&RUN alignment tracks for STAT3 in MAC2A, FE-PD, and JB6 cells, overlaid with H3K27ac ChIP-seq, shown at the gene loci for *BCL2L1* (Bcl-x_L_). Read densities (y-axis) were normalized to reads per million reads sequenced in each sample. **D).** Normalized ChIP-seq alignment track for STAT3 and STAT5 (unstimulated or stimulated with TPO) in the Hematopoietic Pre-Cursor cell line (HPC7), compared to IgG, shown at the gene loci for *Bcl2l1* (Bcl-x_L_). Read densities (y-axis) were normalized to reads per million reads sequenced in each sample. **E).** Representative FACS plots of single stainings (7AAD and Annexin V) used to define gates. **F).** Bar chart depicting % of *fl/fl Pdgfrb* and *Δ/Δ Pdgfrb* cells in either G1 (black), S (light grey) or G2 (dark grey) cell cycle phase. **G).** IC_50_ plots of *fl/fl Pdgfrb* (grey) and *Δ/Δ Pdgfrb* (orange) primary tumor cell lines treated with STAT5 inhibitor AC-4-130 for 72 h with varying concentrations. Mouse embryonic fibroblasts (MEFs) with a knockout of STAT5 were used as a negative control. **A** and **C** Data are shown as mean ± SD, and *p* values were determined by unpaired two-tailed Student’s t-tests (ns = p > 0.05; * = p < 0.05; ** = p < 0.01; *** = p < 0.001; **** = p < 0.0001). **Supplementary Fig. 5. A).** Schematic representation of the individual CRISPR guide RNAs designed to target the genomic murine *Stat5a* locus*, Stat5b* locus and both *Stat5a/b* genes. The sequence of the guide RNA is depicted in blue and the protospacer adjacent motif (PAM) in red. **B).** Western blot showing protein levels of total STAT5 in single clones derived from the bulk population of a *fl/fl Pdgfrb* primary tumor cell line. Efficiency of CRSIPR/Cas9 mediated knockout of both gene products (*Stat5a/b*^*Δ*CRISPR^) was compared to the non-targeting empty control. GAPDH serves as loading control. The molecular weight of analyzed proteins in kiloDaltons (KDa) is shown on the left. Below panel: TIDE assay heatmap. Data represents the percentage of remaining *Stat5a* or *Stat5b* sequence following CRISPR/Cas9 mediated knockout relative to the non-targeting empty control. **C).** Schematic overview of lentiviral transduction using the two-vector system approach. Firstly, *fl/fl Pdgfrb* primary tumor cell lines were lentivirally transduced with the EF1a-Cas9-P2A-EGFP backbone. Cells were next subjected to Fluorescence Activated Cell Sorting for GFP and screened for elevated GFP and Cas9 levels. Once a clone with a stable expression was detected, it was subjected to a second round of lentiviral transduction with the vector system carrying the sgRNAs and mCherry (U6-IT-mPGK-Cherry). Finally, GFP (FITC-A channel) and mCherry (ECD-A channel) expression was measured via CytoFLEX S using ECD-A channel over a period of 39 days post-transduction. **D).** Western blot showing protein levels of total PDGFRβ, total STAT3 and total STAT5 at experimental end point (39-days post transduction). Efficiency of CRSIPR/Cas9 mediated knockout of genes of interest was compared to the non-targeting empty control. GAPDH serves as loading control. The molecular weight of analyzed proteins in kiloDaltons (KDa) is shown on the left. **Supplementary Fig. 6. A).** Schematic representation of AC-4-130 treatment time frame following inoculation of 6 week-old immunocompromised mice. **B).** and **C).** Bar chart depicting body weight in grams (g.) of NSG female mice at 10 days post inoculation with either (*n* = 3) *fl/fl Pdgfrb* and (*n* = 3) *Δ/Δ Pdgfrb* cell lines at experimental end point following vehicle or AC-4-130 treatment in two replicate experiments. **D).** Tumor volume (mm^3^) increase of (*n* = 3) *fl/fl Pdgfrb* and (*n* = 3) *Δ/Δ Pdgfrb* inoculated NSG 6 week-old female mice treated with either vehicle or AC-4-130. **E).** Tumor to body weight ratio in % at experimental end point of NSG mice treated with either (*n* = 3) vehicle or (*n* = 3) AC-4-130. **F).** Western blot showing protein levels of PDGFRβ and Bcl-x_L_ in end point tumors excised from (*n* = 3) *fl/fl Pdgfrb* and (*n* = 3) *Δ/Δ Pdgfrb* inoculated NSG 6 week-old female mice treated with either vehicle or AC-4-130. GAPDH serves as loading control. The molecular weight of analyzed proteins in kiloDaltons (KDa) is shown on the left. PDGFRβ levels over GAPDH and Bcl-x_L_ levels over GAPDH are depicted as relative volume in %. **B, C, E,** and **F** Data are shown as mean ± SD, and *p* values were determined by unpaired two-tailed Student’s t-tests (multiple t-test application on GraphPad) (ns = p > 0.05; * = p < 0.05; ** = p < 0.01; *** = p < 0.001; **** = p < 0.0001).

**Additional file 2.** Supplementary Materials and Methods [32, 34, 40, 61–63].

**Additional file 3: Supplementary Table 1.** Genotyping primers. **Supplementary Table 2.** Antibody list. **Supplementary Table 3.** CUT&RUN and ChIP-Seq Antibodies. **Supplementary Table 4.** Guide RNAs. **Supplementary Table 5.** RT-qPCR primers.

## Data Availability

All data generated or analyzed during the study are included in the manuscript and supplementary information files. The following publicly available datasets were used: the Piccaluga Lymphoma dataset [32] using the Oncomine™ Research Premium Edition database (Thermo Fisher, Ann Arbor, MI) [34] and the human genomic DNA methylation dataset retrieved from Hassler et al.*,* 2016 (data analyzed and tracked on UCSC genome browser). STAT3 human ALK^+^ (JB6) [53] and ALK^−^ (FE-PD and MAC2A) ChIP-Seq data originate from Menotti et al.*,* 2019. Murine STAT3 and STAT5 ChIP-Seq data [54, 60] was obtained from Li et al., 2017 and Liao et al., 2011.

## Abbreviations

ALCL: Anaplastic Large Cell Lymphoma
ALK: Anaplastic Lymphoma Kinase
AML: Acute Myeloid Leukemia
AP-1: Activator Protein-1 Transcription Factor Superfamily
ATP: Adenosine Tri-phosphate
BCL2: B-cell Lymphoma 2
Bcl-x_L_: B-cell Lymphoma Extra-Large
BCR-ABL: Breakpoint Cluster Region-Abelson Murine Leukemia Viral Oncogene
BIA-ALCL: Breast-Implant Associated ALCL
BFM: Berlin-Frankfurt-Münster Chemotherapy regimen
bZIP: Basic Leucine-Zipper Domain
ChIP: Chromatin ImmunoPrecipitation
CHOP: Polychemotherapy: Cyclosphosphoamide, Doxorubicin, Vicristin and Prednisone
CML: Chronic Myelogenous Leukemia
CRE: cAMP Response Element
CRISPR: Clustered Regulatory Interspaced Short Palindromic Repeats
DNA: Deoxyribonucleic Acid
EFS: Event Free Survival
ERK: Extra Cellular Signal-Regulated Kinase
FDA: U.S Food and Drug Administration
FFPE: Fresh Frozen Paraffin Embedded
HEK293: Human Embryonic Kidney Cells
HL: Hodgkin Lymphoma
IL: Interleukin
JAK: Janus Kinase
MEF: Mouse Embryonic Fibroblasts
mTOR: Mammalian Target of Rapamycin
MYB: Myeloblastosis Viral Oncogene Homolog
NHL: Non-Hodgkin Lymphoma
NPM: Nucleophosmin1
NSCLC: Non Small Cell Lung Cancer
NSG: NOD-SCID Il2rg^null^
OS: Overall Survival
PDGFR: Platelet Derived Growth Factor Receptor
PI3K/AKT: Phophatidylinositol-4,5-bisphophate 3-kinase
PTCL: Peripheral T cell Lymphoma
SH2: SRC Homology 2
STAT: Signal Transducers and Activators of Transcription
TKI: Tyrosine Kinase Inhibitor
TYK2: Tyrosine Kinase 2
WHO: World Health Organization

## References

[1] Swerdlow SH, Campo E, Pileri SA (2016). The 2016 revision of the World Health Organization classification of lymphoid neoplasms. Blood..

[2] Stein H, Foss HD, Dürkop H (2000). CD30(+) anaplastic large cell lymphoma: a review of its histopathologic, genetic, and clinical features. Blood..

[3] Morris SW, Kirstein MN, Valentine MB (1994). Fusion of a kinase gene, ALK, to a nucleolar protein gene, NPM, in non-Hodgkin’s lymphoma. Science..

[4] Ducray SP, Natarajan K, Garland GD, Turner SD, Egger G (2019). The transcriptional roles of ALK fusion proteins in tumorigenesis. Cancers (Basel)..

[5] Marzec M, Zhang Q, Goradia A (2008). Oncogenic kinase NPM/ALK induces through STAT3 expression of immunosuppressive protein CD274 (PD-L1, B7-H1). Proc Natl Acad Sci U S A.

[6] Prokoph N, Probst NA, Lee LC (2020). IL10RA modulates crizotinib sensitivity in NPM1-ALK+ anaplastic large cell lymphoma. Blood..

[7] Chiarle R, Simmons WJ, Cai H (2005). Stat3 is required for ALK-mediated lymphomagenesis and provides a possible therapeutic target. Nat Med.

[8] Prutsch N, Gurnhofer E, Suske T (2019). Dependency on the TYK2/STAT1/MCL1 axis in anaplastic large cell lymphoma. Leukemia..

[9] Zhang Q, Wang HY, Liu X, Wasik MA. STAT5A is epigenetically silenced by the tyrosine kinase NPM1-ALK and acts as a tumor suppressor by reciprocally inhibiting NPM1-ALK expression. Nat Med. 2007. 10.1038/nm1659. 10.1038/nm1659 17922009

[10] Brugieres MC, Le Deley H, Pacquement Z (1998). CD30+ Anaplastic Large-Cell Lymphoma in Children: Analysis of 82 Patients Enrolled in Two Consecutive Studies of the French Society of Pediatric Oncology By.

[11] Rosolen A, Pillon M, Garaventa A (2005). Anaplastic large cell lymphoma treated with a leukemia-like therapy: report of the Italian Association of Pediatric Hematology and Oncology (AIEOP) LNH-92 protocol. Cancer..

[12] Seidemann K, Tiemann M, Schrappe M (2001). Short-pulse B-non-Hodgkin lymphoma-type chemotherapy is efficacious treatment for pediatric anaplastic large cell lymphoma: a report of the Berlin-Frankfurt-Münster group trial NHL-BFM 90. Blood..

[13] Laver JH, Kraveka JM, Hutchison RE (2005). Advanced-stage large-cell lymphoma in children and adolescents: results of a randomized trial incorporating intermediate-dose methotrexate and high-dose cytarabine in the maintenance phase of the APO regimen: a pediatric oncology group phase III trial. J Clin Oncol.

[14] Prokoph N, Larose H, Lim MS, Burke GAA, Turner SD (2018). Treatment options for paediatric anaplastic large cell lymphoma (ALCL): current standard and beyond. Cancers (Basel)..

[15] Shaw AT, Kim TM, Crinò L (2017). Ceritinib versus chemotherapy in patients with ALK-rearranged non-small-cell lung cancer previously given chemotherapy and crizotinib (ASCEND-5): a randomised, controlled, open-label, phase 3 trial. Lancet Oncol.

[16] Seto T, Nishio M, Hida T (2019). Final PFS analysis and safety data from the phase III J-ALEX study of alectinib (ALC) vs. crizotinib (CRZ) in ALK-inhibitor naïve ALK-positive non-small cell lung cancer (ALK+ NSCLC). J Clin Oncol.

[17] Camidge DR, Dziadziuszko R, Peters S (2019). Updated Efficacy and Safety Data and Impact of the EML4-ALK Fusion Variant on the Efficacy of Alectinib in Untreated ALK-Positive Advanced Non–Small Cell Lung Cancer in the Global Phase III ALEX Study. J Thorac Oncol.

[18] Huber RM, Hansen KH, Paz-Ares Rodríguez L (2020). Brigatinib in Crizotinib-refractory ALK+ NSCLC: 2-year follow-up on systemic and intracranial outcomes in the phase 2 ALTA trial. J Thorac Oncol.

[19] Camidge DR, Kim HR, Ahn M-J (2020). Brigatinib Versus Crizotinib in Advanced ALK Inhibitor–Naive ALK-Positive Non–Small Cell Lung Cancer: Second Interim Analysis of the Phase III ALTA-1L Trial. J Clin Oncol.

[20] Solomon BJ, Besse B, Bauer TM (2018). Lorlatinib in patients with ALK-positive non-small-cell lung cancer: results from a global phase 2 study. Lancet Oncol..

[21] Drilon A, Siena S, Ou S-HI (2017). Safety and antitumor activity of the multitargeted Pan-TRK, ROS1, and ALK inhibitor Entrectinib: combined results from two phase I trials (ALKA-372-001 and STARTRK-1). Cancer Discov.

[22] Sharma GG, Mota I, Mologni L, Patrucco E, Gambacorti-Passerini C, Chiarle R. Tumor resistance against ALK targeted therapy-where it comes from and where it goes. Cancers (Basel). 2018;10(3). 10.3390/cancers10030062. 10.3390/cancers10030062 PMC5876637 29495603

[23] Gristina V, La Mantia M, Iacono F, Galvano A, Russo A, Bazan V (2020). The emerging therapeutic landscape of ALK inhibitors in non-small cell lung Cancer. Pharmaceuticals..

[24] Gambacorti-Passerini C, Mussolin L, Brugieres L (2016). Abrupt relapse of ALK -positive lymphoma after discontinuation of Crizotinib. N Engl J Med.

[25] Staber PB, Vesely P, Haq N (2007). The oncoprotein NPM-ALK of anaplastic large-cell lymphoma induces JUNB transcription via ERK1/2 and JunB translation via mTOR signaling. Blood..

[26] Laimer D, Dolznig H, Kollmann K (2012). PDGFR blockade is a rational and effective therapy for NPM-ALK-driven lymphomas. Nat Med.

[27] Staber PB, Kornauth C, Garces de los Fayos Alonso I (2019). Imatinib +/− Brentuximab Vedotin induces sustained complete remission in chemotherapy-resistant anaplastic large cell lymphoma expressing PDGFR. Blood..

[28] Chiarle R, Gong JZ, Guasparri I (2003). NPM-ALK transgenic mice spontaneously develop T-cell lymphomas and plasma cell tumors. Blood..

[29] Sawada S (1994). A lineage-specific transcriptional silencer regulates CD4 gene expression during T lymphocyte development. Cell..

[30] Schmahl J, Rizzolo K, Soriano P (2008). The PDGF signaling pathway controls multiple steroid-producing lineages. Genes Dev.

[31] Zimmerman MW, Liu Y, He S (2018). MYC drives a subset of high-risk pediatric neuroblastomas and is activated through mechanisms including enhancer hijacking and focal enhancer amplification. Cancer Discov..

[32] Piccaluga PP, Agostinelli C, Califano A (2007). Gene expression analysis of peripheral T cell lymphoma, unspecified, reveals distinct profiles and new potential therapeutic targets. J Clin Invest.

[33] Redl E, Sheibani-Tezerji R, Cardona CDJ (2021). Requirement of DNMT1 to orchestrate epigenomic reprogramming for NPM-ALK-driven lymphomagenesis. Life Sci Alliance.

[34] Rhodes DR, Yu J, Shanker K (2004). ONCOMINE: a Cancer microarray database and integrated data-mining platform. Neoplasia..

[35] Hassler MR, Pulverer W, Lakshminarasimhan R (2016). Insights into the pathogenesis of anaplastic large-cell lymphoma through genome-wide DNA methylation profiling. Cell Rep.

[36] Zamo A, Chiarle R, Piva R (2002). Anaplastic lymphoma kinase (ALK) activates Stat3 and protects hematopoietic cells from cell death. Oncogene..

[37] Kanai T, Seki S, Jenks JA (2014). Identification of STAT5A and STAT5B Target Genes in Human T Cells. PLoS One.

[38] Kollmann S, Grausenburger R, Klampfl T, et al. A STAT5B-CD9 axis determines self-renewal in hematopoietic and leukemic stem cells. Blood. 2021. 10.1182/blood.2021010980. 10.1182/blood.2021010980 PMC8777465 34320169

[39] Maurer B, Kollmann S, Pickem J, Hoelbl-Kovacic A, Sexl V. STAT5A and STAT5B—twins with different personalities in hematopoiesis and leukemia. Cancers (Basel). 2019;11(11). 10.3390/cancers11111726. 10.3390/cancers11111726 PMC6895831 31690038

[40] Wingelhofer B, Maurer B, Heyes EC, et al. Pharmacologic inhibition of STAT5 in acute myeloid leukemia. Leukemia. 2018. 10.1038/s41375-017-0005-9. 10.1038/s41375-017-0005-9 PMC5940656 29472718

[41] Knörr F, Damm-Welk C, Ruf S (2018). Blood cytokine concentrations in pediatric patients with anaplastic lymphoma kinase-positive anaplastic large cell lymphoma. Haematologica..

[42] Boulland M-L, Meignin V, Leroy-Viard K (1998). Human Interleukin-10 expression in T/natural killer-cell lymphomas. Am J Pathol.

[43] Fielding CA (2012). Interleukin-19: a new target to aim for?. Rheumatology..

[44] Hofmann S, Möller J, Rauen T (2012). Dynamic CpG-DNA methylation of Il10 and Il19 in CD4+ T lymphocytes and macrophages: effects on tissue-specific gene expression. Klin Pädiatrie.

[45] Jordan W, Eskdale J, Boniotto M (2005). Human IL-19 regulates immunity through auto-induction of IL-19 and production of IL-10. Eur J Immunol.

[46] Lobello C, Tichy B, Bystry V (2021). STAT3 and TP53 mutations associate with poor prognosis in anaplastic large cell lymphoma. Leukemia..

[47] Nieborowska-Skorska M, Slupianek A, Xue L (2001). Role of signal transducer and activator of transcription 5 in nucleophosmin/ anaplastic lymphoma kinase-mediated malignant transformation of lymphoid cells. Cancer Res.

[48] Pham HTT, Maurer B, Prchal-Murphy M (2018). STAT5B N642H is a driver mutation for T cell neoplasia. J Clin Invest.

[49] Kollmann S, Grundschober E, Maurer B (2019). Twins with different personalities: STAT5B—but not STAT5A—has a key role in BCR/ABL-induced leukemia. Leukemia..

[50] Maurer B, Nivarthi H, Wingelhofer B (2020). High activation of STAT5A drives peripheral T-cell lymphoma and leukemia. Haematologica..

[51] Wingelhofer B, Neubauer HA, Valent P, et al. Implications of STAT3 and STAT5 signaling on gene regulation and chromatin remodeling in hematopoietic cancer. Leukemia. 2018. 10.1038/s41375-018-0117-x. 10.1038/s41375-018-0117-x PMC6087715 29728695

[52] Orlova A, Wagner C, De Araujo ED (2019). Direct targeting options for STAT3 and STAT5 in cancer. Cancers (Basel)..

[53] Menotti M, Ambrogio C, Cheong T (2019). Wiskott–Aldrich syndrome protein (WASP) is a tumor suppressor in T cell lymphoma. Nat Med.

[54] Li P, Mitra S, Spolski R (2017). STAT5-mediated chromatin interactions in superenhancers activate IL-2 highly inducible genes: functional dissection of the Il2ra gene locus. Proc Natl Acad Sci.

[55] Park HJ, Li J, Hannah R (2016). Cytokine-induced megakaryocytic differentiation is regulated by genome-wide loss of a <scp>uSTAT</scp> transcriptional program. EMBO J.

[56] Jain RK, Lahdenranta J, Fukumura D (2008). Targeting PDGF signaling in carcinoma-associated fibroblasts controls cervical Cancer in mouse model. PLoS Med.

[57] Mathew P, Thall PF, Bucana CD (2007). Platelet-derived growth factor receptor inhibition and chemotherapy for castration-resistant prostate Cancer with bone metastases. Clin Cancer Res.

[58] Mohanty SK, Yagiz K, Pradhan D (2017). STAT3 and STAT5A are potential therapeutic targets in castration-resistant prostate cancer. Oncotarget..

[59] Morgan EL, Macdonald A (2019). JAK2 inhibition impairs proliferation and Sensitises cervical Cancer cells to cisplatin-induced cell death. Cancers (Basel).

[60] Liao W, Lin J-X, Wang L, Li P, Leonard WJ (2011). Modulation of cytokine receptors by IL-2 broadly regulates differentiation into helper T cell lineages. Nat Immunol.

[61] Sanjana NE, Shalem O, Zhang F. Improved vectors and genome-wide libraries for CRISPR screening. Nat Publ Gr. 2014;11. 10.1038/nmeth.3047. 10.1038/nmeth.3047 PMC4486245 25075903

[62] Shah RR, Cholewa-Waclaw J, Davies FCJ, Paton KM, Chaligne R, Heard E, Abbott CM, Bird AP. Efficient and versatile CRISPR engineering of human neurons in culture to model neurological disorders. Wellcome Open Res. 2016;1:13. 10.12688/wellcomeopenres.10011.1. 10.12688/wellcomeopenres.10011.1 PMC5146642 27976757

[63] Lagger S, Meunier D, Mikula M, Brunmeir R, Schlederer M, Artaker M, Pusch O, Egger G, Hagelkruys A, Mikulits W, Weitzer G, Muellner EW, Susani M, Kenner L, Seiser C. Crucial function of histone deacetylase 1 for differentiation of teratomas in mice and humans. EMBO J. 2010;29(23):3992–4007. 10.1038/emboj.2010.264. 10.1038/emboj.2010.264 PMC3020644 20967026

