# Supplementary material for: PDGFRβ promotes oncogenic progression via STAT3/STAT5 hyperactivation in anaplastic large cell lymphoma
Source: Mol Cancer. 2022 Aug 31;21:172. doi: 10.1186/s12943-022-01640-7 (PMC9434917; doi:10.1186/s12943-022-01640-7)

Supplementary Figure 1

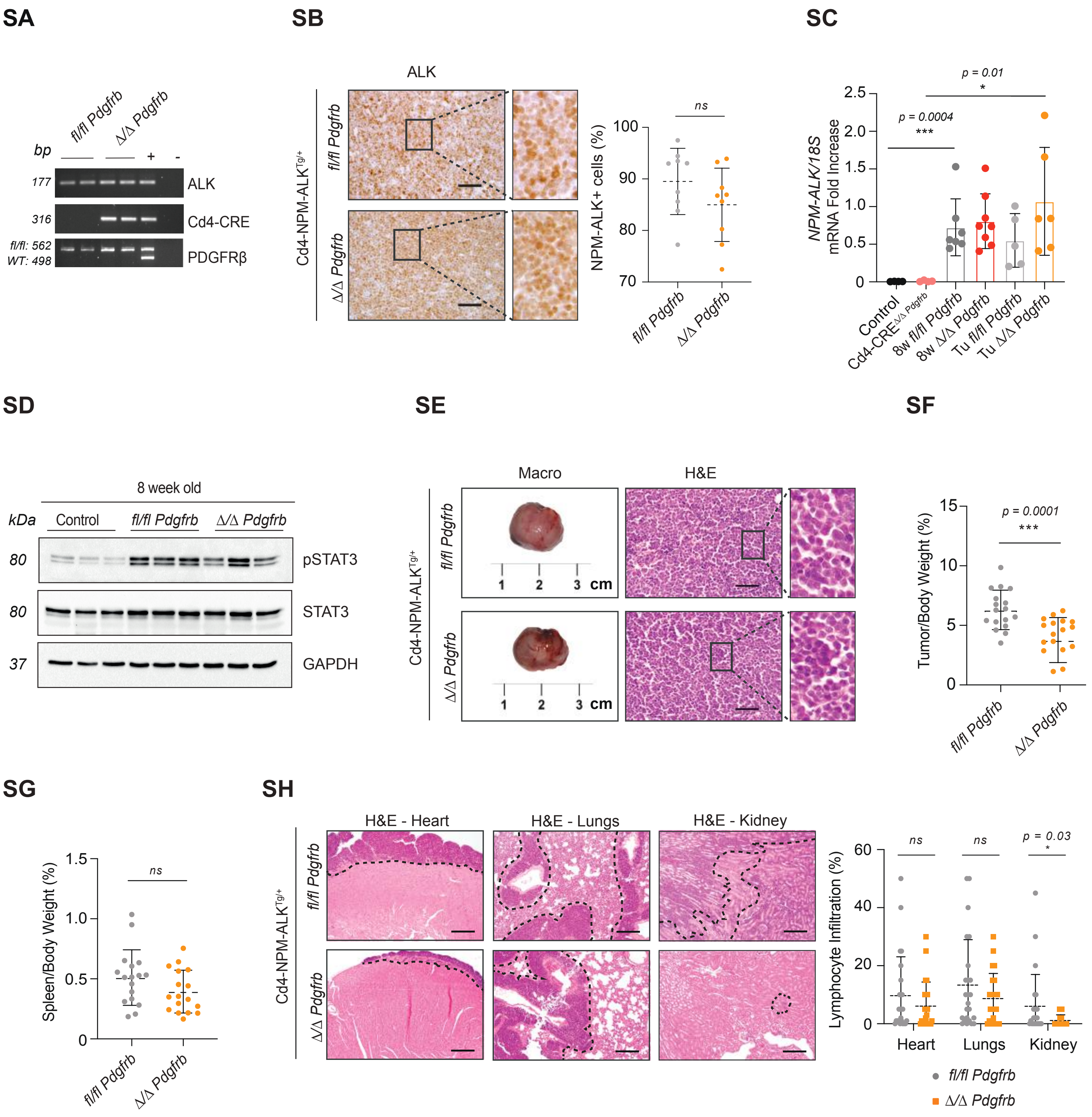

Supplementary Figure 2

SA

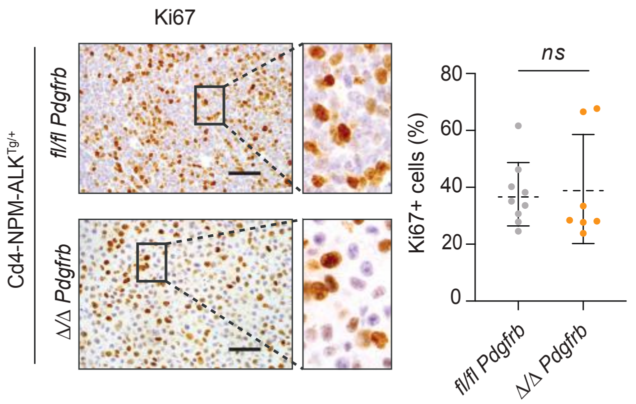

SB

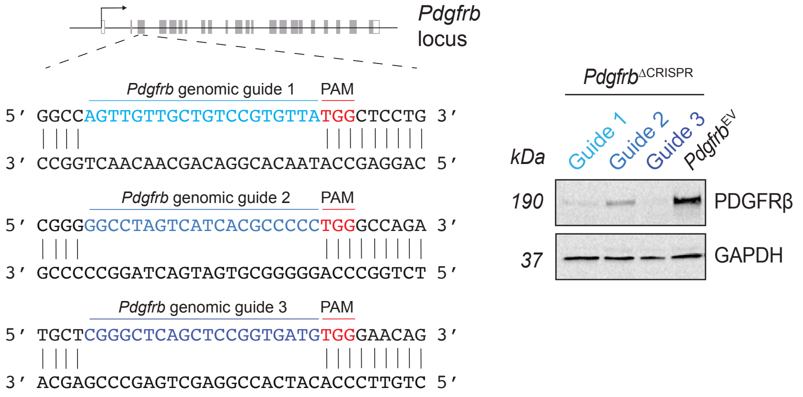

SC

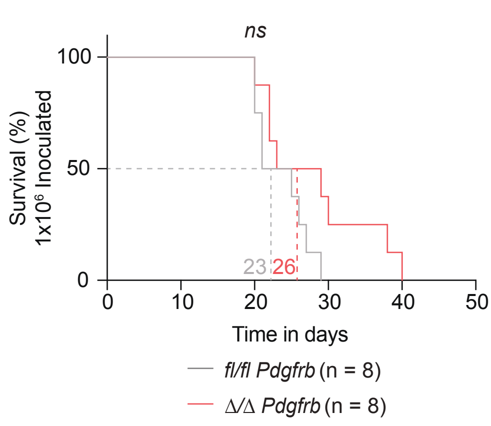

SD

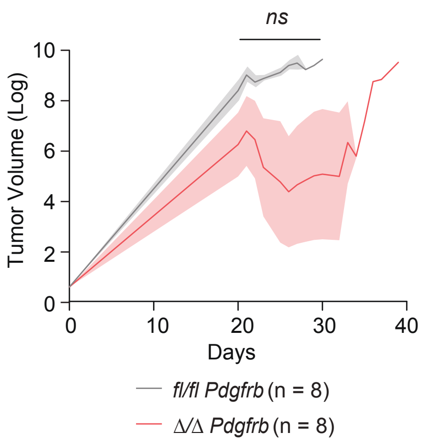

SE

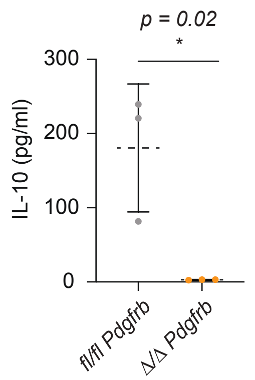

SF

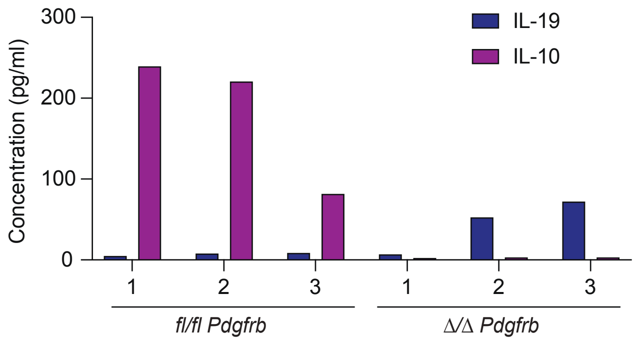

SG

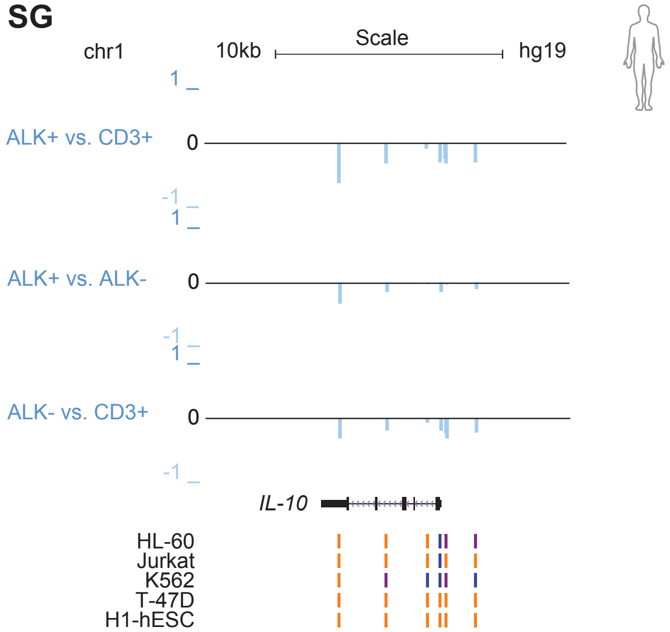

Supplementary Figure 3

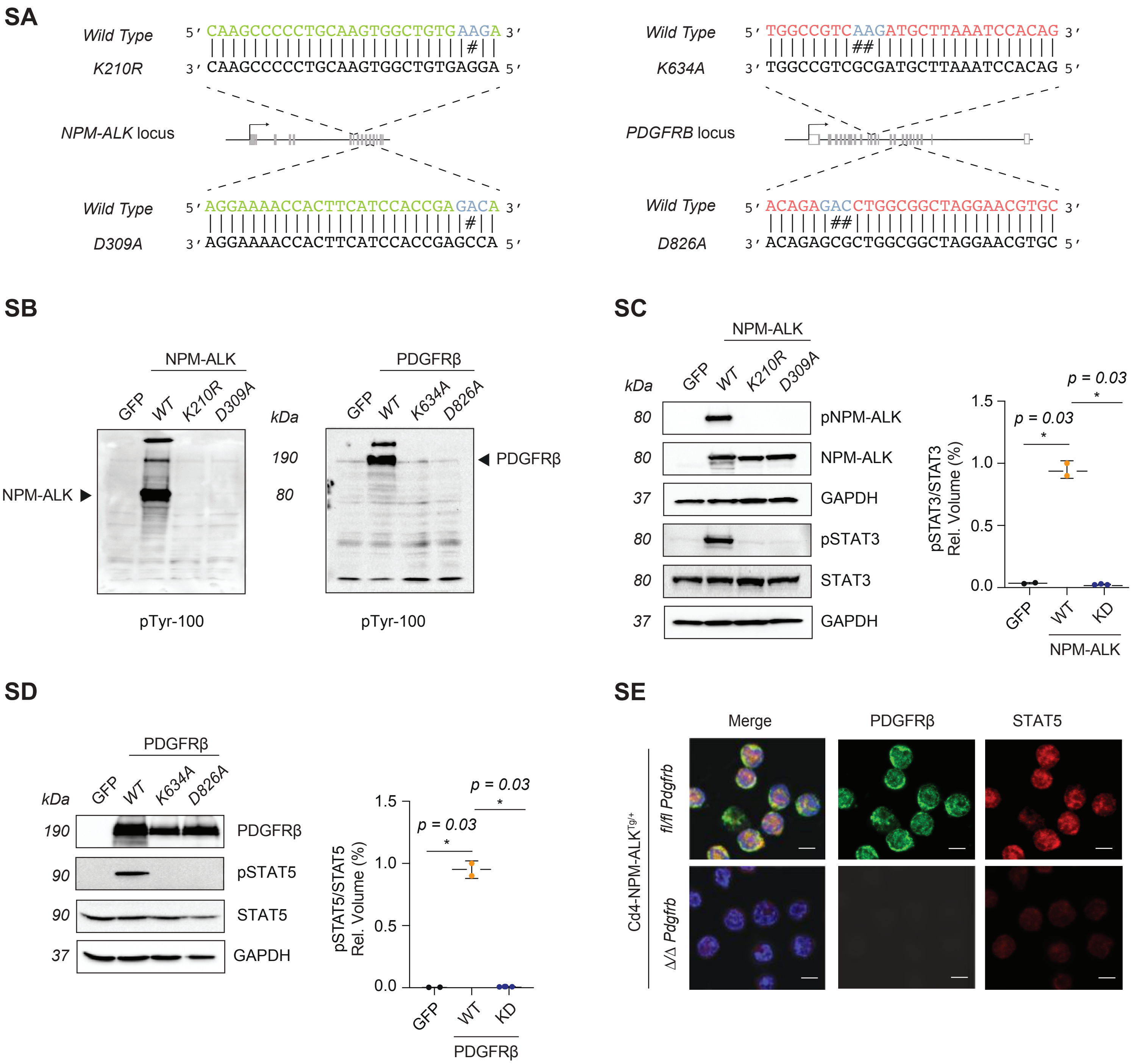

Supplementary Figure 4

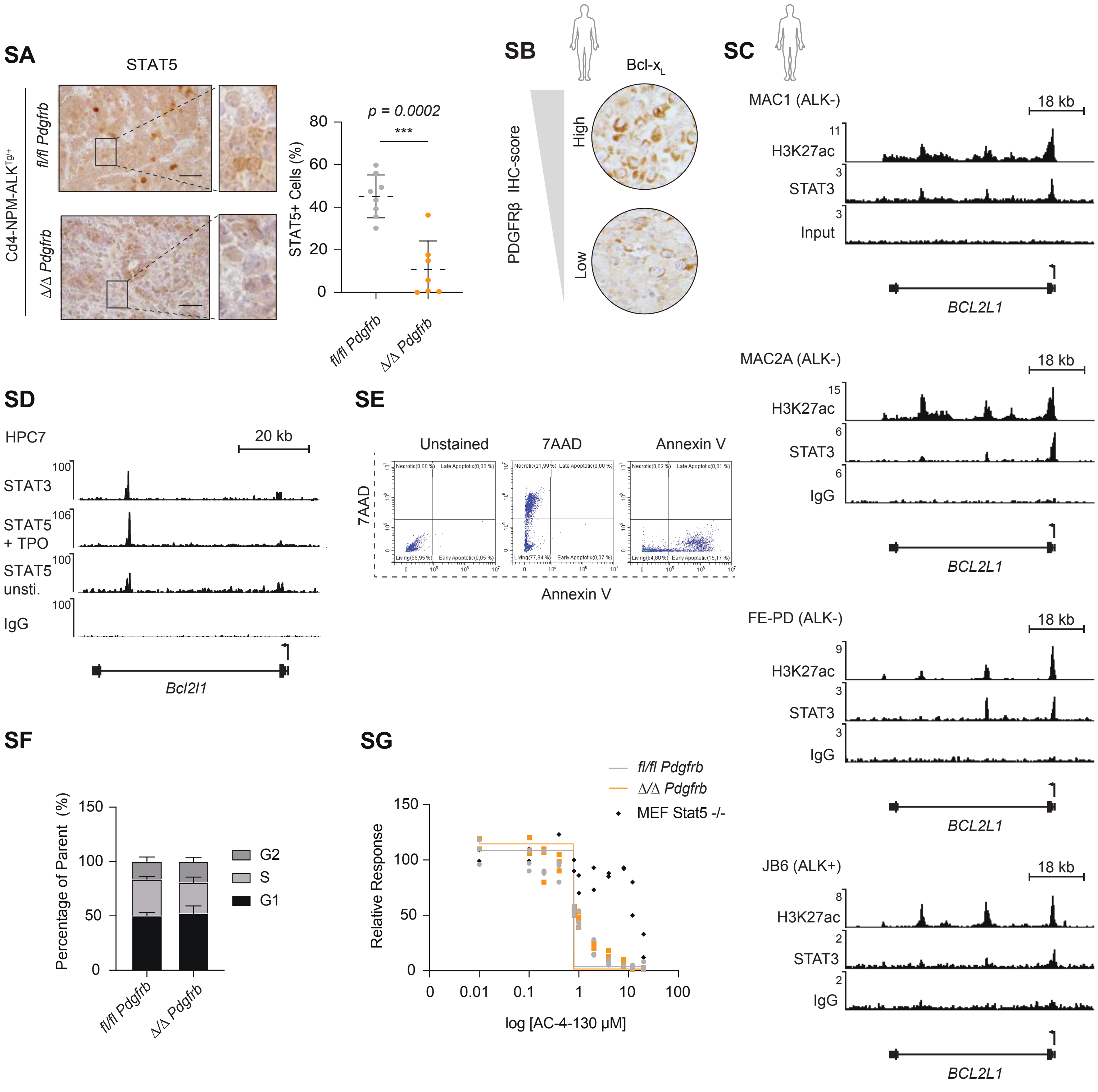

Suplementary Figure 5

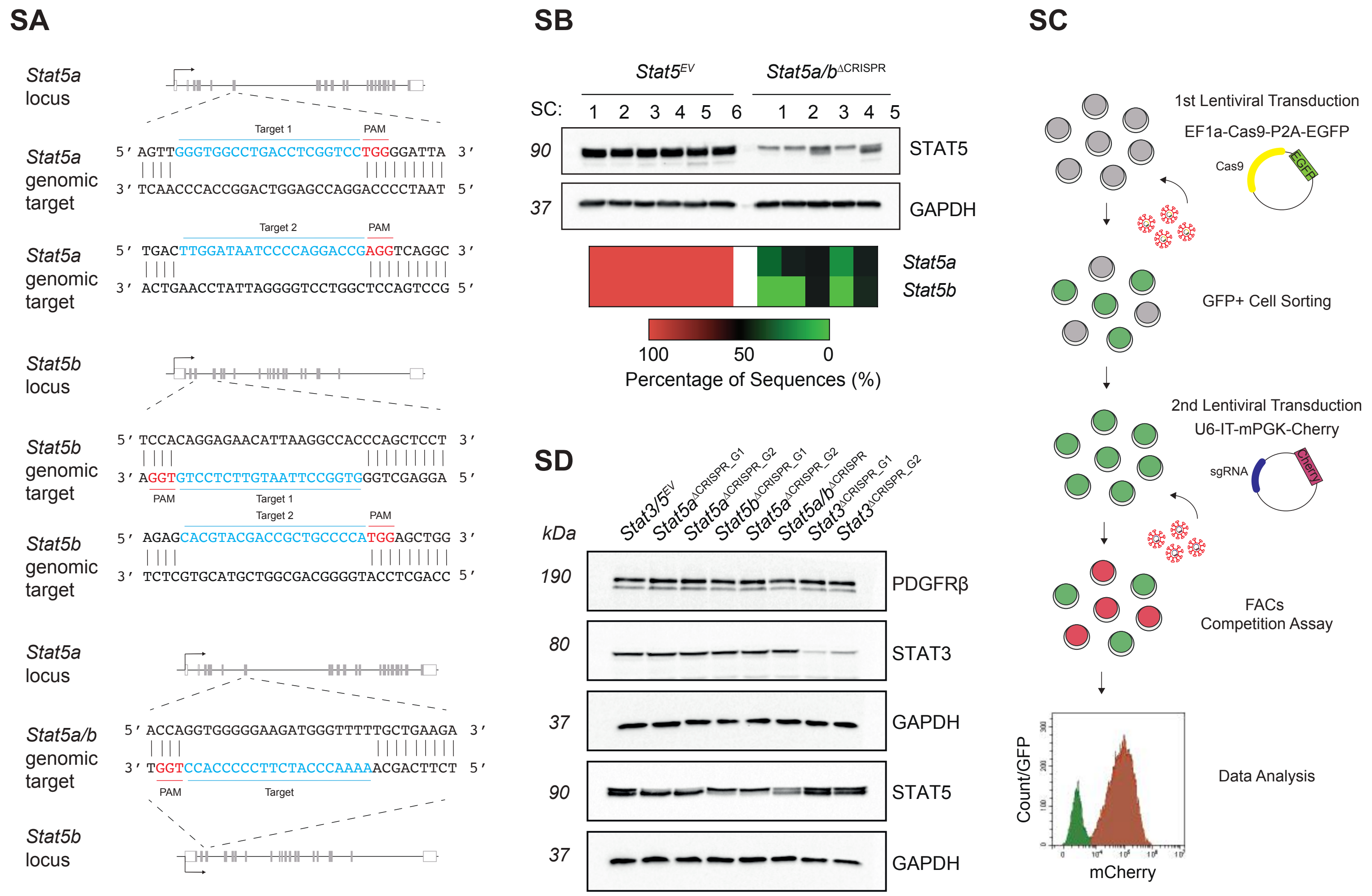

Supplementary Figure 6

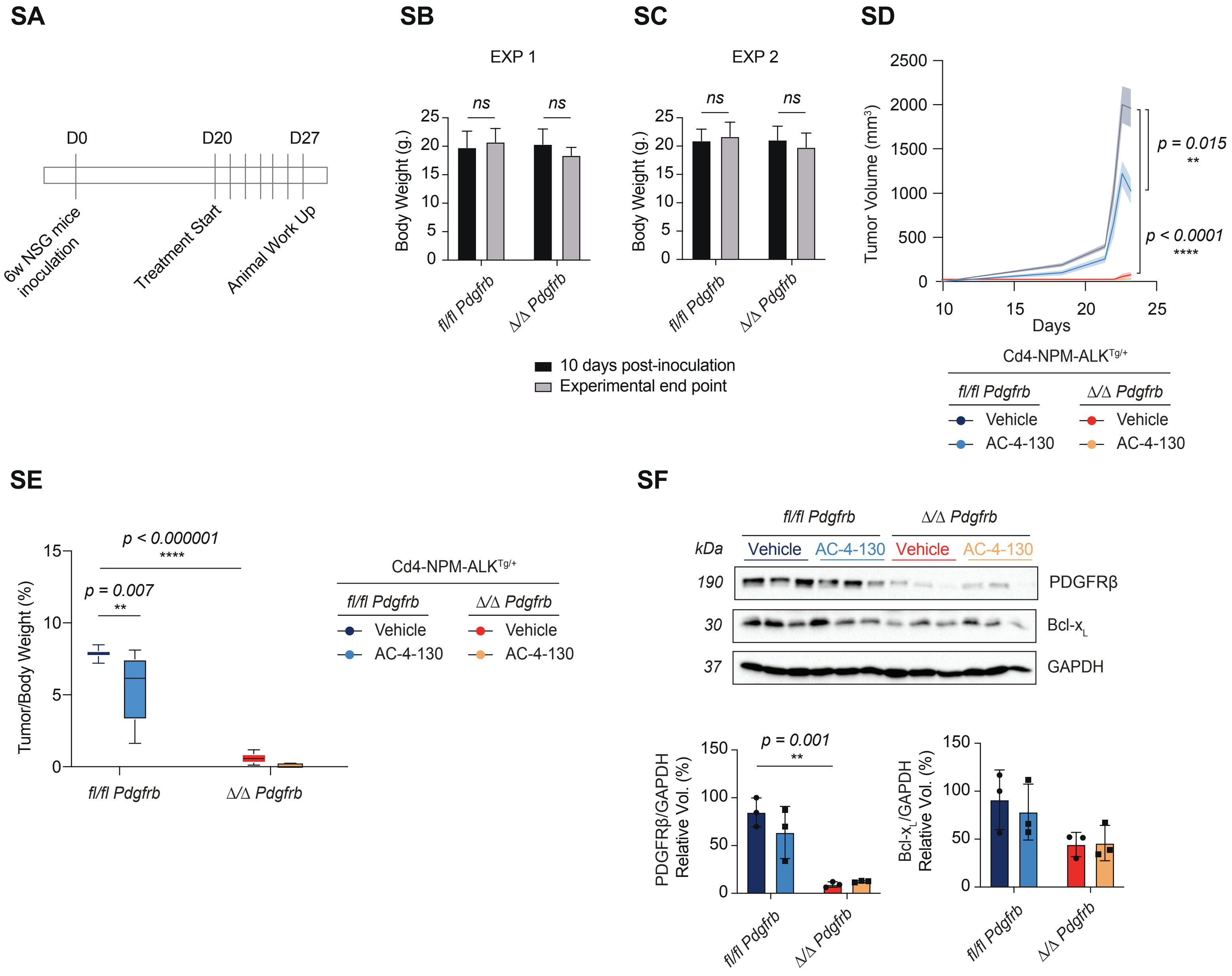

Supplement: Supplementary file 1 — Additional file 1: Supplementary Fig. 1. A). Genotyping PCR from purified genomic mouse tail DNA after Cre expression in fl/fl Pdgfrb and Δ/Δ Pdgfrb samples. Depicted are results for ALK, Cd4-CRE and the Pdgfrb alleles (without loxP sites: wild type (wt); with loxP sites: fl/fl) and the recombined (∆) Pdgfrb locus. Genomic mouse tail DNA from a PDGFRβ fl/+ animal was used as the PDGFRβ genotyping PCR positive control. Expected fragment band sizes: ALK (TG/+: 177 bp), Cd4-CRE (TG/+: 316 bp) and the Pdgfrb (WT: 498 bp and fl/fl: 562 bp). B). Representative pictures of ALK IHC analysis of fl/fl Pdgfrb and Δ/Δ Pdgfrb mouse thymomas at experimental end point. Scale bars: 50 μm. Black squares in the left picture represent the area chosen for the magnification depicted on the right. Stainings of (n = 9) tumor samples per genotype were scanned and whole-slide quantified using the Definiens™ software. C). Quantitative RT-qPCR based quantification of NPM-ALK mRNA transcripts from: (n = 4) control animals (black); (n = 4) Cd4-CREΔ/Δ Pdgfrb lacking NPM-ALK oncogene (pink); (n = 7) fl/fl Pdgfrb 8 week-old developing thymi (dark grey); (n = 6) Δ/Δ Pdgfrb 8 week-old developing thymi (red); (n = 5) end point thymomas (light grey); and (n = 8) end point thymomas (orange). Data was normalized to 18S ribosomal RNA and depicted as a fold-change over one fl/fl Pdgfrb biological replicate set to 1. D). Western blot analysis showing protein levels of phospho (p) STAT3 and total STAT3 for 8 week-old pre-thymoma lysates: (n = 3) control, (n = 3) fl/fl Pdgfrb and (n = 3) Δ/Δ Pdgfrb. GAPDH serves as loading control. The molecular weight of analyzed proteins in kiloDaltons (KDa) is shown on the left. E). Left panel: representative macroscopic pictures of thymomas of fl/fl Pdgfrb and Δ/Δ Pdgfrb mice resected at experimental end point. Scale bar: 3 cm. Right panels: representative H&E-stained sections of tumors from fl/fl Pdgfrb and Δ/Δ Pdgfrb mice at experimental end point. Black squares in the [file 12943_2022_1640_MOESM1_ESM.zip › Revised_Figures_GarcesdelosFayosAlonso_25072022.pdf]
