# Supplementary material for: PDGFRβ promotes oncogenic progression via STAT3/STAT5 hyperactivation in anaplastic large cell lymphoma
Source: Mol Cancer. 2022 Aug 31;21:172. doi: 10.1186/s12943-022-01640-7 (PMC9434917; doi:10.1186/s12943-022-01640-7)
Supplement: Supplementary file 1 — Additional file 1: Supplementary Fig. 1. A). Genotyping PCR from purified genomic mouse tail DNA after Cre expression in fl/fl Pdgfrb and Δ/Δ Pdgfrb samples. Depicted are results for ALK, Cd4-CRE and the Pdgfrb alleles (without loxP sites: wild type (wt); with loxP sites: fl/fl) and the recombined (∆) Pdgfrb locus. Genomic mouse tail DNA from a PDGFRβ fl/+ animal was used as the PDGFRβ genotyping PCR positive control. Expected fragment band sizes: ALK (TG/+: 177 bp), Cd4-CRE (TG/+: 316 bp) and the Pdgfrb (WT: 498 bp and fl/fl: 562 bp). B). Representative pictures of ALK IHC analysis of fl/fl Pdgfrb and Δ/Δ Pdgfrb mouse thymomas at experimental end point. Scale bars: 50 μm. Black squares in the left picture represent the area chosen for the magnification depicted on the right. Stainings of (n = 9) tumor samples per genotype were scanned and whole-slide quantified using the Definiens™ software. C). Quantitative RT-qPCR based quantification of NPM-ALK mRNA transcripts from: (n = 4) control animals (black); (n = 4) Cd4-CREΔ/Δ Pdgfrb lacking NPM-ALK oncogene (pink); (n = 7) fl/fl Pdgfrb 8 week-old developing thymi (dark grey); (n = 6) Δ/Δ Pdgfrb 8 week-old developing thymi (red); (n = 5) end point thymomas (light grey); and (n = 8) end point thymomas (orange). Data was normalized to 18S ribosomal RNA and depicted as a fold-change over one fl/fl Pdgfrb biological replicate set to 1. D). Western blot analysis showing protein levels of phospho (p) STAT3 and total STAT3 for 8 week-old pre-thymoma lysates: (n = 3) control, (n = 3) fl/fl Pdgfrb and (n = 3) Δ/Δ Pdgfrb. GAPDH serves as loading control. The molecular weight of analyzed proteins in kiloDaltons (KDa) is shown on the left. E). Left panel: representative macroscopic pictures of thymomas of fl/fl Pdgfrb and Δ/Δ Pdgfrb mice resected at experimental end point. Scale bar: 3 cm. Right panels: representative H&E-stained sections of tumors from fl/fl Pdgfrb and Δ/Δ Pdgfrb mice at experimental end point. Black squares in the [file 12943_2022_1640_MOESM1_ESM.zip › Supplementary Material Figure Legends.docx]

**Supplementary Material**

**Figure Legends**

**Supplementary Figure 1 –**

**A).** Genotyping PCR from purified genomic mouse tail DNA after *Cre* expression in *fl/fl Pdgfrb* and *Δ/Δ Pdgfrb* samples. Depicted are results for *ALK*, *Cd4*-*CRE* and the *Pdgfrb* alleles (without *loxP* sites: wild type (wt); with *loxP* sites: *fl*/*fl*) and the recombined (∆) *Pdgfrb* locus. Genomic mouse tail DNA from a PDGFRβ fl/+ animal was used as the PDGFRβ genotyping PCR positive control. Expected fragment band sizes: *ALK* (TG/+: 177 bp), *Cd4*-*CRE* (TG/+: 316 bp) and the *Pdgfrb* (WT: 498 bp and fl/fl: 562 bp). **B).** Representative pictures of ALK IHC analysis of *fl/fl Pdgfrb* and *Δ/Δ Pdgfrb* mouse thymomas at experimental end point. Scale bars: 50 µm. Black squares in the left picture represent the area chosen for the magnification depicted on the right. Stainings of (n = 9) tumor samples per genotype were scanned and whole-slide quantified using the Definiens^TM^ software. **C).** Quantitative RT-qPCR based quantification of *NPM-ALK* mRNA transcripts from: (n = 4) control animals (black); (n = 4) *Cd4*-CRE*^Δ/Δ Pdgfrb^* lacking NPM-ALK oncogene (pink); (n = 7) *fl/fl Pdgfrb* 8 week-old developing thymi (dark grey); (n = 6) *Δ/Δ Pdgfrb* 8 week-old developing thymi (red) ; (n = 5) end point thymomas (light grey); and (n = 8) end point thymomas (orange). Data was normalized to *18S* ribosomal RNA and depicted as a fold-change over one *fl/fl Pdgfrb* biological replicate set to 1. **D).** Western blot analysis showing protein levels of phospho (p) STAT3 and total STAT3 for 8 week-old pre-thymoma lysates: (n = 3) control, (n = 3) *fl/fl Pdgfrb* and (n = 3) *Δ/Δ Pdgfrb*. GAPDH serves as loading control. The molecular weight of analyzed proteins in kiloDaltons (KDa) is shown on the left. **E).** Left panel: representative macroscopic pictures of thymomas of *fl/fl Pdgfrb* and *Δ/Δ Pdgfrb* mice resected at experimental end point. Scale bar: 3cm. Right panels: representative H&E-stained sections of tumors from *fl/fl Pdgfrb* and *Δ/Δ Pdgfrb* mice at experimental end point*.* Black squares in the middle picture represent the area chosen for the magnification depicted on the right. Scale bars: 50 µm. **F).** Thymic tumor weight from (n = 17) *fl/fl Pdgfrb* (grey) and (n = 17) *Δ/Δ Pdgfrb* (orange) mice normalized to whole body weight at experimental end point. **G).** Spleen weight from (n = 17) *fl/fl Pdgfrb* (grey) and (n = 17) *Δ/Δ Pdgfrb* (orange) mice normalized to whole body weight at experimental end point. **H).** H&E-stained sections of tumor dissemination into the kidney, heart and lungs from *fl/fl Pdgfrb* and *Δ/Δ Pdgfrb* mice at experimental end point*.* Black dashed line represents malignant infiltration into secondary organ. Scale bars: 50 µm. Individual biological replicates used for statistical analyzes are shown in the graph below. **B, C, F, G** and **H** Data are shown as mean ± SD, and *p* values were determined by unpaired two-tailed Student’s t-tests (*ns = p > 0.05; * = p < 0.05; ** = p < 0.01; *** = p < 0.001; **** = p < 0.0001*).

**Supplementary Figure 2 –**

**A).** Representative pictures of Ki67 IHC analysis of (n = 9) *fl/fl Pdgfrb* and (n = 7) *Δ/Δ Pdgfrb* murine thymomas at experimental end point. Black squares in the left pictures represent the area chosen for the magnification depicted on the right. Scale bars: 50 µm. Whole-slide scans were quantified using the Definiens software. **B).** Schematic representation of three individual CRISPR guide RNAs designed to target the genomic murine *Pdgfrb* locus. The sequence of the guide RNA is depicted in blue and the protospacer adjacent motif (PAM) in red. Right panel: Western blot showing protein levels of PDGFRβ in three *fl/fl Pdgfrb* primary tumor cell lines subjected to CRISPR/Cas9 deletion of *Pdgfrb* (*Pdgfrb*^ΔCRISPR^) and empty vector transduced control (*Pdgfrb^EV^)*. GAPDH serves as loading control. The molecular weight of analyzed proteins in kiloDaltons (KDa) is shown on the left. **C).** Kaplan Meier cumulative survival analysis of 6 week-old NSG females inoculated with either (n = 8) *fl/fl Pdgfrb* (grey) or (n = 8) *Δ/Δ Pdgfrb* (orange) primary mouse tumor cell lines at a high concentration (1 x10^6^ cells/flank). Values next to the dotted lines on the x-axis indicate median life expectancy before tumor size reaches the 2000 mm^3^ set threshold volume. **D).** Longitudinal analysis of tumor volume increase of 6 week-old NSG female mice inoculated with either (n = 8) *fl/fl Pdgfrb* (grey) or (n = 8) *Δ/Δ Pdgfrb* (orange) primary mouse tumor cell lines at a high concentration (1 x10^6^ cells/ flank). **E).** Cytokine assay measuring IL-10 concentration (pg/ml) of (n = 3) *fl/fl Pdgfrb* (grey) and (n = 3) *Δ/Δ Pdgfrb* (orange) primary mouse tumor cell lines. **F).** Bar chart depicting IL-10 (red) and IL-19 (blue) concentration (pg/ml) in the supernatant of (n = 3) *fl/fl Pdgfrb* and (n = 3) *Δ/Δ Pdgfrb* primary mouse tumor cell lines seeded at a low density (1 x10^5^). **G).** Differential genomic DNA methylation on the human *IL-10* locus. Top panel: ALK^+^ (n = 5) and ALK^-^ (n = 5) ALCL patient samples compared to (n = 5) healthy CD3^+^ T cells. Data were retrieved from Hassler *et al.,* 2016. Middle panel: UCSC gene annotation track indicating *IL-10* gene. Lower panel: CpG Methylation obtained from Methyl 450K Bead Arrays from ENCODE/HAIB depicting HL-60, Jurkat, K562, T-47D and H1-hESC human cell lines. Orange: methylated (score > = 600), Purple: partially methylated (200 < score < 600), Bright Blue: unmethylated (0 < score < = 200). **A** and **E** Data are shown as mean ± SD and *p* values were determined by unpaired two-tailed Student’s t-tests. **C** *p* value was determined by log-rank (Mantel-Cox) test (*ns = p > 0.05; * = p < 0.05; ** = p < 0.01; *** = p < 0.001; **** = p < 0.0001*).

**Supplementary Figure 3 –**

**A).** Schematic representation of the wild type and mutated kinase dead versions of either NPM-ALK (green) or PDGFRβ (red). Exons depicted in grey, altered amino acids in blue and hashtag represents mutated nucleotide. **B).** Western blot showing phosphotyrosine-100 (pTyr-100) levels in HEK293FT cells transfected with either wild type or kinase dead versions of PDGFRβ or NPM-ALK. **C).** Western blot analysis showing protein levels of phospho (p) NPM-ALK, total NPM-ALK, phospho (p) STAT3 and total STAT3 in HEK293FT transfected cell lysates. GAPDH serves as loading control. The molecular weight of analyzed proteins in kiloDaltons (KDa) is indicated. **D).** Western blot showing protein levels of PDGFRβ, phospho (p) STAT5 and total STAT5 in HEK293FT transfected cell lysates. GAPDH serves as loading control. The molecular weight of analyzed proteins in kiloDaltons (KDa) is shown on the left. Phospho (p) STAT5 levels over total STAT5 are depicted as relative volume in %. **E).** Double immunofluorescence staining of *fl/fl Pdgfrb* and *Δ/Δ Pdgfrb* primary tumor cells derived from our transgenic Cd4-NPM-ALKTg/+ model. Cells were fixed and stained with antibodies against PDGFRβ (green) and STAT5 (red). Cells were counterstained with DAPI (blue). Pictures were acquired with identical pixel density, image resolution, and exposure time using a confocal LSM Observer Z.1 Zeiss Microscope. Scale bars: 5 µm. **C** and **D** Data are shown as mean ± SD and *p* values were determined by unpaired two-tailed Student’s t-tests (*ns = p > 0.05; * = p < 0.05; ** = p < 0.01; *** = p < 0.001; **** = p < 0.0001*).

**Supplementary Figure 4 –**

**A).** Representative pictures of total STAT5 IHC analysis of (n = 8) *fl/fl Pdgfrb* and (n = 7) *Δ/Δ Pdgfrb* mouse thymomas at experimental end point. Black squares in the left pictures represent the area chosen for the magnification depicted on the right. Scale bars: 50 µm. Whole-slide scans were quantified using the Definiens^TM^ software (right graph). **B).** Representative pictures of Bcl-x_L_ IHC analysis and the PDGFRβ grading scheme used to quantify staining of tissue microarrays from human ALCL tumor samples. **C).** Normalized ChIP-seq alignment track for STAT3 in MAC1 cells, and normalized CUT&RUN alignment tracks for STAT3 in MAC2A, FE-PD, and JB6 cells, overlaid with H3K27ac ChIP-seq, shown at the gene loci for *BCL2L1* (Bcl-x_L_). Read densities (y-axis) were normalized to reads per million reads sequenced in each sample. **D).** Normalized ChIP-seq alignment track for STAT3 and STAT5 (unstimulated or stimulated with TPO) in the Hematopoietic Pre-Cursor cell line (HPC7), compared to IgG, shown at the gene loci for *Bcl2l1* (Bcl-x_L_). Read densities (y-axis) were normalized to reads per million reads sequenced in each sample. **E).** Representative FACS plots of single stainings (7AAD and Annexin V) used to define gates. **F).** Bar chart depicting % of *fl/fl Pdgfrb* and *Δ/Δ Pdgfrb* cells in either G1 (black), S (light grey) or G2 (dark grey) cell cycle phase. **G).** IC_50_ plots of *fl/fl Pdgfrb* (grey) and *Δ/Δ Pdgfrb* (orange) primary tumor cell lines treated with STAT5 inhibitor AC-4-130 for 72h with varying concentrations. Mouse embryonic fibroblasts (MEFs) with a knockout of STAT5 were used as a negative control. **A** and **C** Data are shown as mean ± SD, and *p* values were determined by unpaired two-tailed Student’s t-tests (*ns = p > 0.05; * = p < 0.05; ** = p < 0.01; *** = p < 0.001; **** = p < 0.0001*).

**Supplementary Figure 5 –**

**A).** Schematic representation of the individual CRISPR guide RNAs designed to target the genomic murine *Stat5a* locus*, Stat5b* locus and both *Stat5a/b* genes. The sequence of the guide RNA is depicted in blue and the protospacer adjacent motif (PAM) in red. **B).** Western blot showing protein levels of total STAT5 in single clones derived from the bulk population of a *fl/fl Pdgfrb* primary tumor cell line. Efficiency of CRSIPR/Cas9 mediated knockout of both gene products (*Stat5a/b^Δ^*^CRISPR^) was compared to the non-targeting empty control. GAPDH serves as loading control. The molecular weight of analyzed proteins in kiloDaltons (KDa) is shown on the left. Below panel: TIDE assay heatmap. Data represents the percentage of remaining *Stat5a* or *Stat5b* sequence following CRISPR/Cas9 mediated knockout relative to the non-targeting empty control. **C).** Schematic overview of lentiviral transduction using the two-vector system approach. Firstly, *fl/fl Pdgfrb* primary tumor cell lines were lentivirally transduced with the EF1a-Cas9-P2A-EGFP backbone. Cells were next subjected to Fluorescence Activated Cell Sorting for GFP and screened for elevated GFP and Cas9 levels. Once a clone with a stable expression was detected, it was subjected to a second round of lentiviral transduction with the vector system carrying the sgRNAs and mCherry (U6-IT-mPGK-Cherry). Finally, GFP (FITC-A channel) and mCherry (ECD-A channel) expression was measured *via* CytoFLEX S using ECD-A channel over a period of 39 days post-transduction. **D).** Western blot showing protein levels of total PDGFRβ, total STAT3 and total STAT5 at experimental end point (39-days post transduction). Efficiency of CRSIPR/Cas9 mediated knockout of genes of interest was compared to the non-targeting empty control. GAPDH serves as loading control. The molecular weight of analyzed proteins in kiloDaltons (KDa) is shown on the left.

**Supplementary Figure 6 –**

**A).** Schematic representation of AC-4-130 treatment time frame following inoculation of 6 week-old immunocompromised mice. **B).** and **C).** Bar chart depicting body weight in grams (g.) of NSG female mice at 10 days post inoculation with either (n = 3) *fl/fl Pdgfrb* and (n = 3) *Δ/Δ Pdgfrb* cell lines at experimental end point following vehicle or AC-4-130 treatment in two replicate experiments. **D).** Tumor volume (mm^3^) increase of (n = 3) *fl/fl Pdgfrb* and (n = 3) *Δ/Δ Pdgfrb* inoculated NSG 6 week-old female mice treated with either vehicle or AC-4-130. **E).** Tumor to body weight ratio in % at experimental end point of NSG mice treated with either (n = 3) vehicle or (n = 3) AC-4-130. **F).** Western blot showing protein levels of PDGFRβ and Bcl-x_L_ in end point tumors excised from (n = 3) *fl/fl Pdgfrb* and (n = 3) *Δ/Δ Pdgfrb* inoculated NSG 6 week-old female mice treated with either vehicle or AC-4-130. GAPDH serves as loading control. The molecular weight of analyzed proteins in kiloDaltons (KDa) is shown on the left. PDGFRβ levels over GAPDH and Bcl-x_L_ levels over GAPDH are depicted as relative volume in %. **B, C, E,** and **F** Data are shown as mean ± SD, and *p* values were determined by unpaired two-tailed Student’s t-tests (multiple t-test application on GraphPad) (*ns = p > 0.05; * = p < 0.05; ** = p < 0.01; *** = p < 0.001; **** = p < 0.0001*).
