# Supplementary material for: PDGFRβ promotes oncogenic progression via STAT3/STAT5 hyperactivation in anaplastic large cell lymphoma
Source: Mol Cancer. 2022 Aug 31;21:172. doi: 10.1186/s12943-022-01640-7 (PMC9434917; doi:10.1186/s12943-022-01640-7)
Supplement: Supplementary file 2 — Additional file 2. Supplementary Materials and Methods [32, 34, 40, 61–63]. [file 12943_2022_1640_MOESM2_ESM.docx]

**Supplementary Materials and Methods**

**Allograft experiments and statistical analysis**

Primary murine tumor cells derived from *fl/fl Pdgfrb* and *Δ/Δ Pdgfrb* animals were inoculated into immunodeficient mice (NOD.Cg-*Prkdc^scid^*, *Il2rd^tm1Wjl^*/SzJ). 6 week-old female mice were maintained under pathogen-free conditions at the Medical University of Vienna. All animal experiments were carried out according to the animal license protocols (BMWFW-66.009 /0183-WF/V/3b/2017; BMWFW-66.009/0057-V/3b/2018; BMWFW-66.009/0401-V/3b/2018; 2020-0.103.412) approved by the ethics committee of the Medical University of Vienna and the Austrian Federal Ministry of Science and Research. The experimental design and number of mice assigned to each cohort was based on prior experience and statistical modelling to insure sufficient power to discern a significant difference amongst the cohorts. No mice were excluded from the analysis.

*Density dependent growth:* 6 week-old NOD.Cg-*Prkdc^scid^*, *Il2rd^tm1Wjl^*/SzJ mice were inoculated with either low (1x10^5^) or ten-fold higher (1x10^6^) densities of cells derived from *fl/fl Pdgfrb* or *Δ/Δ Pdgfrb* animals. Primary murine tumor cells were implanted subcutaneously in both flanks in 100µl of PBS using a 27G needle. Tumor growth was monitored daily using Vernier calipers for the duration of the treatment. Tumor volumes were calculated as the product of length x width x height. Animals were sacrificed when the tumor volume reached 2000 mm^3^ in size. Upon experimental termination, tumors were resected and used for analysis of tumor weight, IHC and WB as described.

*AC-4-130 in vivo treatment:* 6 week-old NOD.Cg-*Prkdc^scid^*, *Il2rd^tm1Wjl^*/SzJ mice were inoculated with 1x10^5^ cells derived from *fl/fl Pdgfrb* or *Δ/Δ Pdgfrb* animals. The animals were randomly divided into two groups and treated daily with either AC-4-130 (25 mg/kg) or vehicle (10% DMSO, 5% Cremophore in saline buffer) by intraperitoneal injection, as previously described^39^.

*Statistical analysis:* Longitudinal analyzes of tumor growth curves over the duration of the experiment was performed using the open-access R package *TumGrowth*. Bonferroni analysis did not reveal any outliers and thus all animals were used for a type II ANOVA and pairwise comparisons across the groups.

**Oncomine database analysis**

Gene expression data for *PDGFRB* (reporter: 202273_at)*, PDGFRA* (reporter: 1554828_at)*, BCL2L1* (reporter: 206665_s_at)*, IL-10* (reporter: 207433_at)*, BCL2* (reporter: 203685_at)*, MCL1* (reporter: 200796_s_at) and *IL19* (reporter: 220745_at) were extracted from the Piccaluga Lymphoma dataset^32^ using the Oncomine™ Research Premium Edition database (Thermo Fisher, Ann Arbor, MI)^59^.

**Cell culture and viability assays**

Primary tumor cells isolated from *fl/fl Pdgfrb* and *Δ/Δ Pdgfrb* were grown in RPMI 1640 Medium (Sigma-Aldrich) supplemented with 10% Fetal Bovine Serum (FBS), 100 U/mL penicillin, and 100 μg/mL penicillin/streptomycin (Gibco). HEK293FT cells were maintained in Dulbecco’s modified Eagle medium supplemented with 10% FBS and 100 μg/mL penicillin/ streptomycin. Cell lines were tested for mycoplasma contamination using the MycoAlert Detection Kit (Lonza) according to manufacturer’s instructions.

1x10^5^ cells were seeded in 6-well plates for proliferation assay and 1x10^4^ cells were seeded into 96-well plates for limiting dilution growth assays. Cell concentrations and viabilities were determined *via* Trypan Blue (Biozym) incorporation or Resazurin (Sigma) and counted with a Luna-II™ Automated Cell Counter (Logos Biosystems).

**Constructs, viral particle production and cell transduction**

# *CRISPR/Cas9 Genome Editing:* CRISPR guide RNAs targeting the genomic DNA of mouse *Pdgfrb* (ENSMUSG00000024620), *Stat5a* (ENSMUST00000004145.14), *Stat5b* (ENSMUST00000107358.9), *Stat5a/b (*ENSMUST00000004145.14 and ENSMUST00000107358.9), *Stat3* (ENSMUST00000127638.8), *Rosa* and *Myb* (kindly gifted by the Grebien Lab) (Suppl. Table 4), were designed using the Zhang lab online design tool (www.crispr.mit.edu), and were cloned into LentiCRISPR v2 and pRRL-U6-sgRNA-IT-PGK-Cherry according to the protocol available from Addgene^61^. The guide RNAs were tested for cutting efficiency with a T7 Endonuclease I assay (NEB) as previously described (Shah et al., 2016).

# *One vector approach:* LentiCRISPR v2 was a gift from Feng Zhang (Addgene plasmid #52961; <http://n2t.net/addgene:52961>; RRID: Addgene_52961).

# *Two vector approach:* pRRL-U6-sgRNA-IT-PGK-Cherry plasmid (kindly gifted by the Zuber Lab, IMP Vienna) and the LentiCRISPRv2GFP (Addgene plasmid #82416; <https://www.addgene.org/82416/>; RRID: Addgene_82416).

# *Virus production:* Generation of viral supernatants was done as described previously^62^. Briefly, lentiviral particles were produced by co-transfecting constructs with packaging and envelope plasmids psPAX2 and pMD2.G in human embryonic kidney cells (HEK293FT) seeded in 10 cm tissue culture dishes using Lipofectamine 2000 (Life Technologies). psPAX2 was a gift from Didier Trono (Addgene plasmid #12260; <http://n2t.net/addgene:12260>; RRID: Addgene_11260). pMD2.G was a gift from Didier Trono (Addgene plasmid #12259; <http://n2t.net/addgene:12259>; RRID: Addgene_11259). 72 hours post-transfection, viral supernatants were harvested, and virus concentrated using the PEG virus precipitation kit (BioVision, Inc), divided into aliquots, snap-frozen in liquid nitrogen and stored at -80°C.

# *Transduction and selection:* 5x10^5^ cells were transduced with 30 μL of viral supernatant in the presence of 10 μg/mL hexadimethrine bromide (Sigma) in 6-well plates for 72 hours. Cells were selected with 2 μg/mL puromycin for 48 hours.

# CRISPR/Cas9-based Competition Assay

# Murine *fl/fl Pdgfrb* and *Δ/Δ Pdgfrb* cells were lentivirally transduced with EF1a-Cas9-P2A-EGFP (kindly gifted by the Zuber Lab, IMP Vienna) and FACS sorted for GFP into 96-well plates. Following the recovery of the single clones, cells were screened for GFP and Cas9 expression. A clone with equal high expression of both GFP and Cas9 was selected for the second lentiviral transduction with the pRRL-U6-sgRNA-IT-PGK-Cherry vector carrying either *Stat5a*, *Stat5b*, *Stat3*, *Myb* or *Rosa* sgRNAs. The dynamics of GFP or mCherry levels were monitored until 39 days post transduction. Data was normalized to values on day 9 post transduction and the non-targeting control *Rosa* sgRNA. Data was analyzed and plotted using the FACS Diva Software (Becton-Dickinson).

# Flow cytometry analysis

# Single-cell suspensions were analyzed with a BD FACS Canto II flow cytometer equipped with 488, 633 and 405 nm lasers using FACS Diva Software (Becton-Dickinson). Analysis of apoptotic cells was conducted according to the manufacturer’s instructions (Annexin V Apoptosis Detection Kit eFluor^®^ 450, eBioscience) using 1x10^5^ cells. Cell cycle analysis was performed by staining 1x10^5^ ethanol-fixed cells with PI (50 μg/ml) in a hypotonic lysis solution (0.1% sodium citrate, 0.1% Triton X-100 and 100 μg/mL RNAse) following incubation at 37°C for 30 minutes.

# Immunofluorescence staining

# Cells were washed with PBS before seeding onto adhesion slides (Marienfeld). Following a pre-extraction with 0.1% Tween in PBS (PBST), cells were fixed with 2% PFA in PBS and permeabilized using 0.5% Triton in PBS. Cells were blocked with 5% BSA in PBST prior to overnight primary antibody incubation at 4°C (Suppl. Table 2). Secondary antibody incubation was performed for 1h at room temperature. Secondary antibodies were diluted accordingly, 1:2000 Alexa Fluor 546 goat anti-mouse (Invitrogen #A11018) and 1:2000 Alexa Fluor 488 goat anti-rabbit (Invitrogen #A11070). 0.2 μg/mL DAPI in PBS was used to counterstain the cells prior to mounting with DAKO (Agilent Technologies) medium. Slides were imaged using a LSM 700 laser scanning confocal microscope (Carl Zeiss).

# TIDE assay

# Tracking of Indels by DEcomposition (TIDE) assay was performed to validate the frequencies and spectrum of insertions or deletions following CRISPR/Cas9 genome editing. Genomic DNA was isolated from snap frozen cell pellets of *Stat5a/b^Δ^*^CRISPR^ and *Stat5a/b^EV^* single clones, using Quick-DNA Miniprep Plus Kit (D4068; Zymo Research) according to the manufacturer’s protocol. Amplification primers were designed to isolate the individual CRISPR target sites using Q5^®^ High - Fidelity DNA Polymerase (M0491S; NEB). Following Spectrophotometer measurement of concentrations, purified amplicons were mixed with the TIDE Sequencing Primer, designed to bind 200 bp upstream of the sgRNA target site. Empty vector single clones were sequenced with both sequencing primers as control sequences. Sequences were further analyzed *via* TIDE Software provided as a free web service <http://shinyapps.datacurators.nl/tide/> (Brinkman et al., 2014). Amplification primers: *Stat5a:* *5'*-*CCCTGAGTCTTCACCTCCAG-3’* and *Stat5b: 5'*-*AGGGAAGGGAGGAAGGAGTA-3’.* TIDE sequencing primers: *Stat5a:* *5'*-*AAGGGGAAGAGGGAAACTGG-3’* and *Stat5b: 5'*-*AATCCCAGCACCCACAAAAC -3’.*

# Construction of expression plasmids and site-directed mutagenesis

# Template generation: Full-length human PDGFRβ cDNA sequences (Sino Biological) was amplified using the Q5 High-Fidelity DNA Polymerase (NEB) and cloned into the pLKO.1 vector harboring the Cytomegalovirus (CMV) promoter and a puromycin resistance, *via* *EcoRI* and *SalI* restriction sites. Similarly, NPM-ALK cDNA was cloned into a modified pLKO.1 vector harboring the CMV promoter and a neomycin resistance, *via* *EcoRI* and *SalI* restriction sites. pLKO.1 was a gift from Bob Weinberg (Addgene plasmid #8453; <https://www.addgene.org/8453/>; RRID: Addgene_8453) and the resistance-cassette was modified by Oliver Pusch.

# Site Directed Mutagenesis: The kinase-dead constructs of NPM-ALK were generated by mutating the K_210_ residue to an arginine or the D_309_ residue to an alanine. For generation of PDGFRβ kinase-dead constructs, the ATP site L_634_ reside and the active site D_826_ residue were separately mutated to an alanine. The sense primers for NPM-ALK K_210_R: 5'-*CTTCAGGCAGCGTCCTCACAGCCACTTGC*-3'; NPM-ALK D_309_A: *5'-GTTTCTGGCAGCAATGGCTCGGTGGATGAAGTG-3',* PDGFRβ L_634_A: *5'-GGCTGTGGATTTAAGCATCGCGACGGCCACTTTCATCGTG-3'*, PDGFRβ D_826_A: *5'*-*CGTTCCTAGCCGCCGCGTCTCTGTGGACGC*-*3'*, were designed using the open QuickChange Primer Design online tool. The mutant constructs of PDGFRβ and NPM-ALK were generated using the QuickChange II XL Site-Directed mutagenesis (Agilent) according to the kit instructions. Full-length PDGFRβ and NPM-ALK cloned into the pLKO.1 vector were used as the template for the respective kinase-dead mutants. Mutations were confirmed by sequencing of the mutant expression vector.

# Protein isolation and immunoblotting

# Cells or tissues were lysed with Hunt Buffer (20 mM Tris-HCl pH 8.0, 100 mM NaCl, 1 mM EDTA, 0.5% w/v NP-40) containing phosphatase and protease inhibitors (COmplete, Roche) using a freeze-thaw method. Equivalent amounts of proteins (20-40 μg) were diluted in sample buffer and separated by SDS-PAGE gels. Proteins were transferred onto nitrocellulose membranes (Protran, Whatman), subjected to immunoblot analysis and stained with antibodies listed in Suppl. Table 2. Anti-mouse (VWR #NXA931) and anti-rabbit (Cell Signaling #7074) HRP conjugated secondary antibodies were used. Chemiluminescent visualization was performed with a ChemiDoc™ Imaging System (Bio-Rad) after incubation of the membranes with Clarity Western ECL reagent (Bio-Rad). Quantifications were performed with Image Lab software (Bio-Rad).

# Quantitative RT-PCR

# Total RNA from murine tumor samples or cell lines was extracted using TRIzol™ reagent (Invitrogen) as instructed by the manufacturer. Following a DNAseI digest, RNA was isolated using the RNA Clean and Concentrator kit (Zymo Research). Total RNA (1 μg) was reverse transcribed using the iScript cDNA synthesis kit (Bio-Rad) and diluted 1:5. Quantitative PCR analysis was performed using the 2x Blue S’Green qPCR mastermix (Biozym). Specific primers for genes of interest designed using Primer3 (Suppl. Table 5).

# IC_50_ determination and inhibitor treatments

# For determining 50% inhibitory concentrations (IC_50_) cells were cultured in 96-well plates for 72 hours in a range of concentrations of AC-4-130 in DMSO, or DMSO as a vehicle control. Cell numbers were measured using resazurin and the signal intensity was measured using a plate reader. Normalized values were plotted on GraphPad and used to obtain survival curves and IC_50_ values.
