# Supplementary material for: PDGFRβ promotes oncogenic progression via STAT3/STAT5 hyperactivation in anaplastic large cell lymphoma
Source: Mol Cancer. 2022 Aug 31;21:172. doi: 10.1186/s12943-022-01640-7 (PMC9434917; doi:10.1186/s12943-022-01640-7)
Supplement: Supplementary file 3 — Additional file 3: Supplementary Table 1. Genotyping primers. Supplementary Table 2. Antibody list. Supplementary Table 3. CUT&RUN and ChIP-Seq Antibodies. Supplementary Table 4. Guide RNAs. Supplementary Table 5. RT-qPCR primers. [file 12943_2022_1640_MOESM3_ESM.docx]

# Table 1 – Genotyping primers

| **Primer ID** | | **Sequence (5’>3’)** | **Annealing Temp.** | **Fragment Size** |
| --- | --- | --- | --- | --- |
| NPM-ALK | Fw | *TCC CTT GGG GGC TTT GAA ATA ACA CC* | 58°C | TG/+ 177 bp |
|  | Rv | *CGA GGT GCG GAG CTT GCT CAG C* |  |  |
| Pdgfrb | Fw | *TAA TCC GAT GCC TTC TGT CC* | 57°C | WT: 498 bp  HZG: 562 bp and 498 bp  FL: 562 bp |
|  | Rv | *TAC CAG GAA GGC TTG GGA AG* |  |  |
| Cd4-CRE | Fw | *ATG CTT CTG TCC GTT TGC CG* | 58°C | TG/+ 316 bp |
|  | Rv | *TGA GTG AAC GAA CCT GGT CG* |  |  |

**Table 2 – Antibody list**

| **Primary Antibody** | **Source** | **Size (kDA)** | **CAT Number** | **Dilution and Application** |
| --- | --- | --- | --- | --- |
| Phospho (p)STAT3 | *rabbit* | *79, 86* | *CST#9145S* | WB: 1:1000 |
| Total STAT3 | *mouse* | *79, 86* | *CST#9139S* | WB: 1:1000 |
| Phospho (p)STAT5 | *rabbit* | *90* | *CST#9359S* | WB: 1:500 |
| Total STAT5 | *rabbit* | *90* | *CST#94205S* | WB: 1:1000  IHC: 1:150 |
| Total STAT5 | *mouse* | *90* | *AF#2168* | IF: 1:200 |
| Phospho (p)ALK | *rabbit* | *80* | *CST#3341S* | WB: 1:1000 |
| Total ALK | *rabbit* | *80* | *CST#3633S* | WB: 1:1000  IHC: 1:250 |
| PDGFRβ | *rabbit* | *190* | *CST#3169S* | WB: 1:1000  IHC: 1:150  IF: 1:200 |
| GAPDH | *rabbit* | *37* | *CST#5174S* | WB: 1:2500 |
| Bcl-x_L_ | *rabbit* | *30* | *CST#2764S* | WB: 1:1000  IHC: 1:5000 |
| CC3 | *rabbit* | *17,19* | *CST#9664S* | WB: 1:1000  IHC: 1:400 |
| Ki67 | *rabbit* | *359* | *CST#12202* | IHC: 1:1000 |
| Phospho (p)-Tyrosine 100 (pTyr-100) | *mouse* |  | *CST#9411* | WB: 1:1000 |

# Table 3 – CUT&RUN and ChIP-Seq Antibodies

| **Cut & Run** | **Manufacturer** | **Catalog** | **Lot** |
| --- | --- | --- | --- |
| STAT3 (D3Z2G) | Cell Signaling | 126403 | 4 |
| Mouse IgG | Santa Cruz Biotechnology | sc-2025 | G2314 |
|  |  |  |  |
| **ChIP-seq** | **Manufacturer** | **Catalog** | **Lot** |
| STAT3 (D3Z2G) | Cell Signaling | 126403 | 4 |
| H3K27ac | Abcam | ab4729 | GR3251519-1 |

# Table 4 – Guide RNAs

| **Guide ID** | **Sequence (5’>3’)** |
| --- | --- |
| *mPdgfrb_guide_1* | *GGCCTAGTCATCACGCCCCC* |
| *mPdgfrb_guide_2* | *GAGTTGTTGCTGTCCGTGTTA* |
| *mPdgfrb_guide_3* | *GCGGGCTCAGCTCCGGTGATG* |
| *mStat5a_guide_1* | *CACCGGGGTGGCCTGACCTCGGTCC* |
| *mStat5a_guide_2* | *CACCGTTGGATAATCCCCAGGACCG* |
| *mStat5b_guide_1* | *CACCG GTGGCCTTAATGTTCTCCTG* |
| *mStat5b_guide_2* | *CACCGCACGTACGACCGCTGCCCCA* |
| *mStat5a/b_guide* | *CACCGAAAACCCATCTTCCCCCACC* |
| *mStat3_guide_1* | *CACCGGCAGCTGGACACACGCTACC* |
| *mStat3_guide_2* | *CACCGGGAAGCTGTCGCTGTACAGC* |
| *mRosa_guide* | *GAAGATGGGCGGGAGTCTTC* |
| *mMyb_guide* | *GAAGCTGGTGGAACAGAA* |

**Table 5 – RT-qPCR primers**

| **Primer ID** | **Forward Sequence (5’>3’)** | **Reverse Sequence (5’>3’)** |
| --- | --- | --- |
| NPM-ALK | *GTT CAG GGC CAG TGC ATA TT* | *TTG GGG TTG TAG TCG GTC AT* |
| Pdgfrb | *AAC AGA AGA CAG CGA GGT GG* | *TGG TAT CAC TCC TGG AAG CC* |
| IL-10 | *AAG CTC CAA GAC CAA GGT GT* | *AGC TCT GTC TAG GTC CTG GA* |
| 18S rRNA | *GCC CGA AGC GTT TAC TTT GA* | *TCC ATT ATT CCT AGC TGC GGT ATCa* |
